# Supplementary material for: Nuclear myosin VI maintains replication fork stability
Source: Nat Commun. 2023 Jun 24;14:3787. doi: 10.1038/s41467-023-39517-y (PMC10290672; doi:10.1038/s41467-023-39517-y)

**SUPPLEMENTARY INFORMATION**

**Nuclear myosin VI maintains replication fork stability**


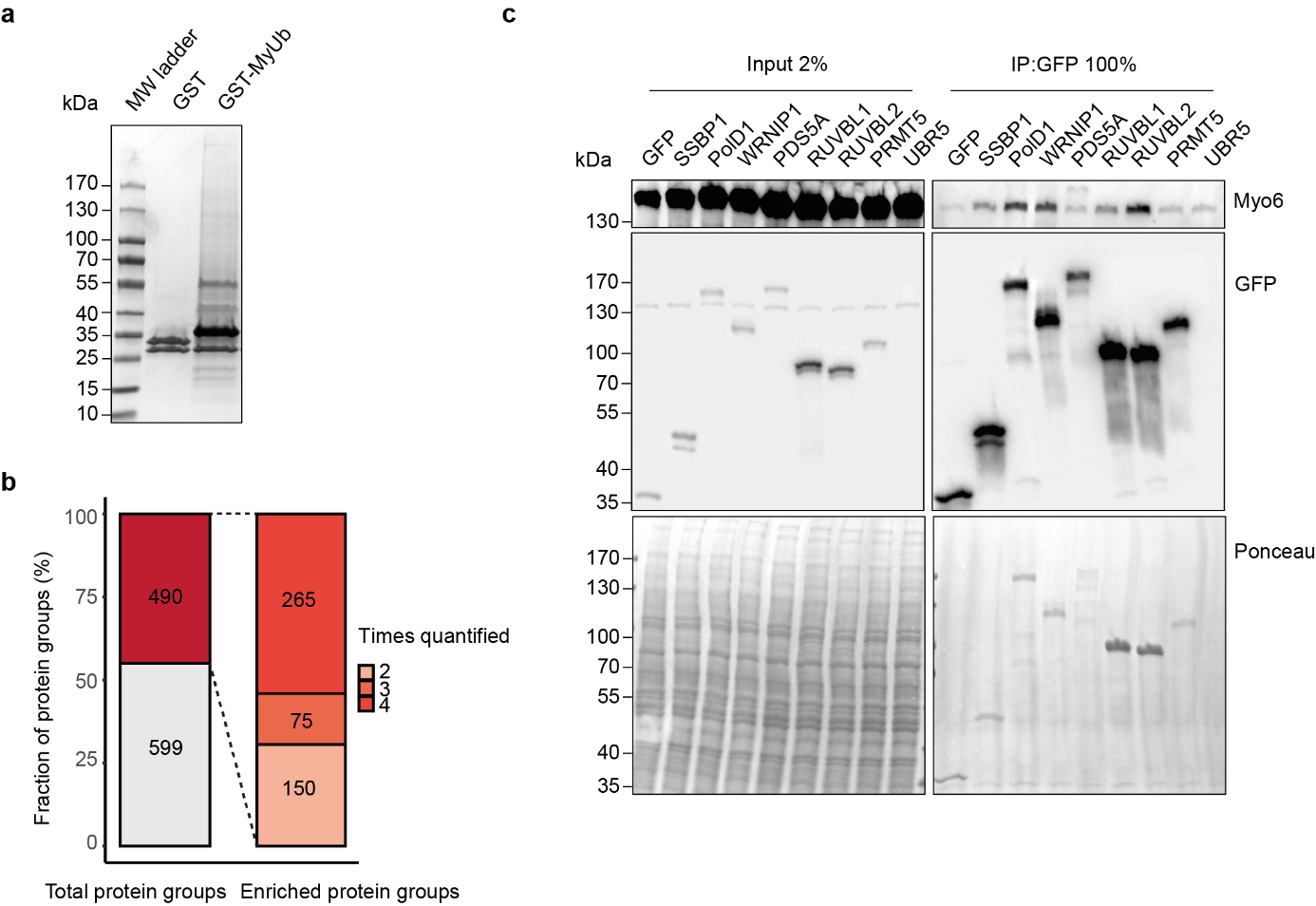


**Supplementary Fig. 1 Identification of novel Myosin VI interactors**

**a**, GST-pulldown assay using GST or GST-MyUb as bait and SILAC-labeled cellular lysates as prey. Samples were separated via SDS-PAGE and proteins were stained using a Colloidal Blue staining kit.

**b**, Left: Bar plot showing the number of significantly enriched protein groups in the pulldown using GST-MyUb followed by LC-MS/MS (fold change > 2, FDR < 0.05). Right: Number of replicates in which the enriched proteins were quantified.

**c,** Validation of novel interactors. HEK293T cells were transfected with GFP fusion constructs of the indicated proteins for 24 h. Immunoprecipitations (IPs) against GFP using GFP-trap beads (Chromotek) were performed, followed by western blotting with antibodies against myosin VI and GFP. Ponceau S staining was performed to control for equal loading. For panels **a** and **c**: Results were confirmed by at least two independent experiments. Uncropped blots are shown at the end of this file.

**
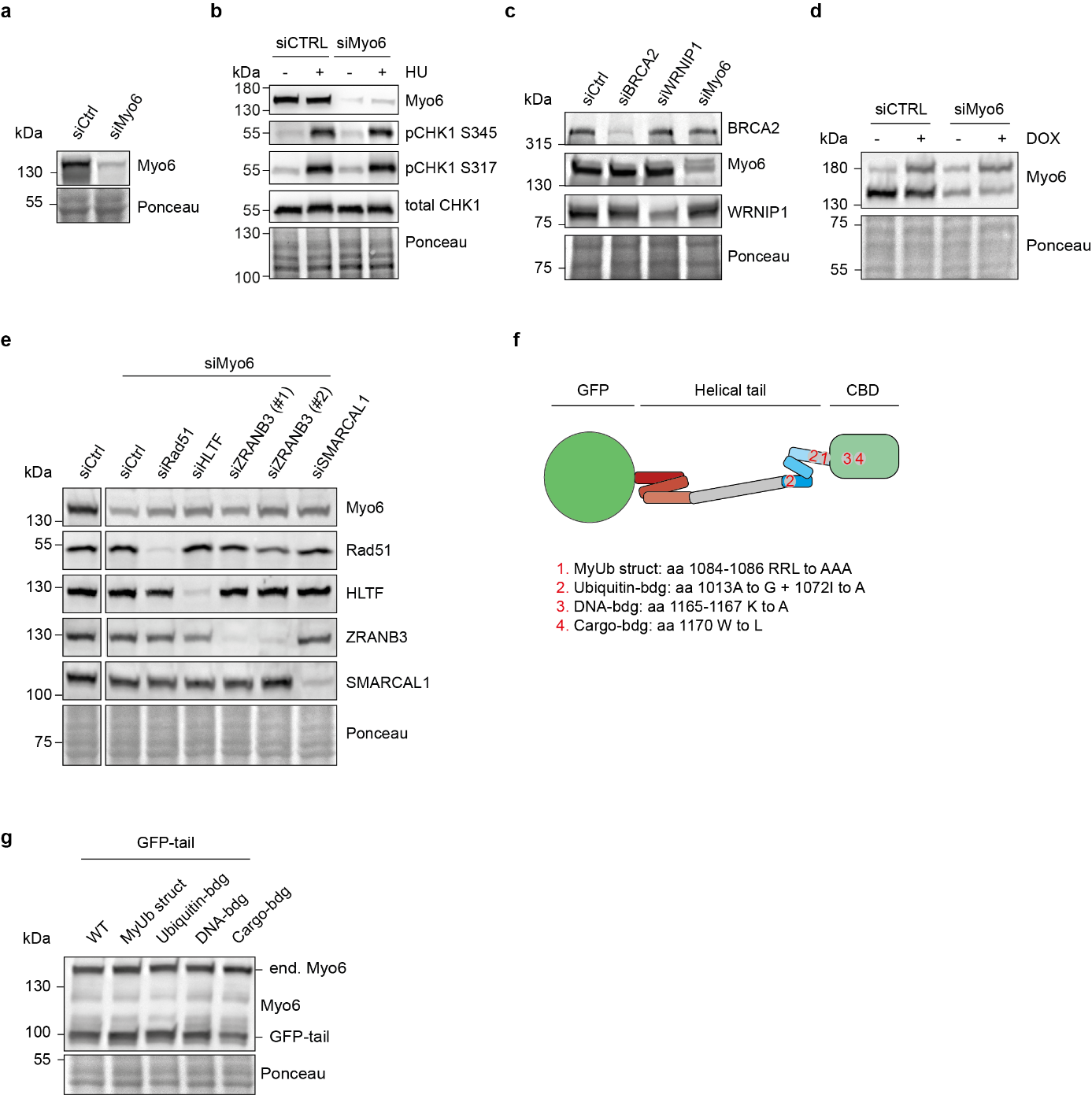
**

**Supplementary Fig. 2 Knockdown efficiencies in fiber assay experiments shown in Fig. 2**

**a**,**c**,**d**,**e**,**g**, Western blots to monitor knockdown efficiencies and expression levels for the fiber assays shown in **Fig. 2**. U2OS cells were transfected with the indicated siRNAs for 72 h or with the indicated overexpression constructs for 48 h, followed by western blotting using the indicated antibodies. Ponceau S staining was performed to control for equal loading. Panel **a** relates to Fig. **2a**, panel **c** relates to Fig. **2b**, panel **d** relates to Fig. **2c**, panel **e** relates to Fig. **2d**, panel **g** relates to Fig. **2e**.

**b**, Depletion of myosin VI does not induce ATR activation. U2OS cells were transfected with the indicated siRNAs for 72 h and treated with 4 mM HU for 1 h as indicated, followed by western blotting using the indicated antibodies. Ponceau S staining was performed to control for equal loading.

**f**, Schematic representation of the respective GFP-tail mutants according to Fig. **1a** used in panel **g**.

For panels **a-e** and **g:** Results were confirmed by at least three independent experiments. Uncropped blots are shown at the end of this file.


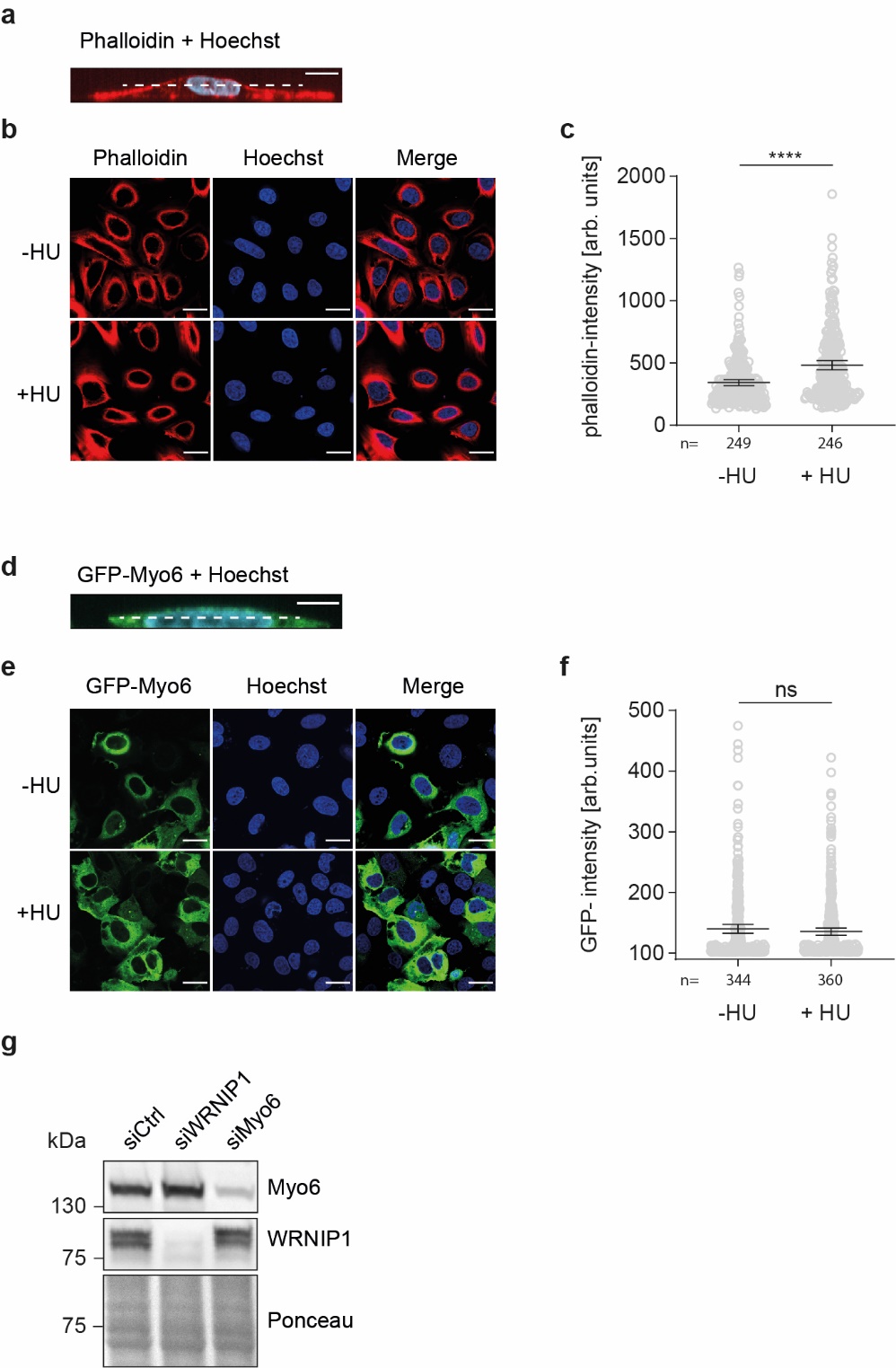


**Supplementary Fig. 3 Nuclear actin and myosin VI levels upon HU treatment**

**a-c**, Confocal microscopy shows an increase in nuclear F-actin upon HU-induced replication stress. U2OS cells were treated for 5 h with 4 mM HU where indicated, followed by staining with Hoechst and Alexa Fluor 647 Phalloidin. A central plane (dashed line) was chosen for quantification of nuclear F-actin **(a)**, scale bar 50 µm. Example images are shown in panel **b**, scale bar 25 µm. Relative nuclear intensities from the phalloidin channel were quantified from the indicated number of cells using Image J and plotted with mean values -/+ 95% confidence intervals. Significance levels in panel **c** were calculated using the two-tailed Mann-Whitney test (****: p<0.0001).

**d-f**, Confocal microscopy shows no difference in nuclear myosin VI levels upon HU-induced replication stress. Note that the detection of endogenous nuclear myosin VI via confocal microscopy was not possible with the available antibodies. Instead, we visualized ectopically expressed myosin VI. U2OS cells were transfected with GFP-Myo6 for 24 h and treated for 5 h with 4 mM HU where indicated, followed by staining with Hoechst. A central plane (dashed line) was chosen for quantification of nuclear GFP-Myo6 **(d)**, scale bar 50 µm. Example images are shown in panel **e**, scale bar 25 µm. Relative nuclear intensities from the GFP channel (488 nm) were quantified from the indicated number of cells using Image J and plotted with mean values -/+ 95% confidence intervals. Significance levels in panel **f** were calculated using the two-tailed Mann-Whitney test (ns: non-significant).

**g**, Western blots to monitor knockdown efficiencies for the fiber assay shown in Fig. **3h**. U2OS cells were transfected with the indicated siRNAs for 72 h, followed by western blotting using the indicated antibodies. Ponceau S staining was performed to control for equal loading. Uncropped blots are shown at the end of this file.

For panels **a-g**: Results were confirmed by at least three independent experiments.

**
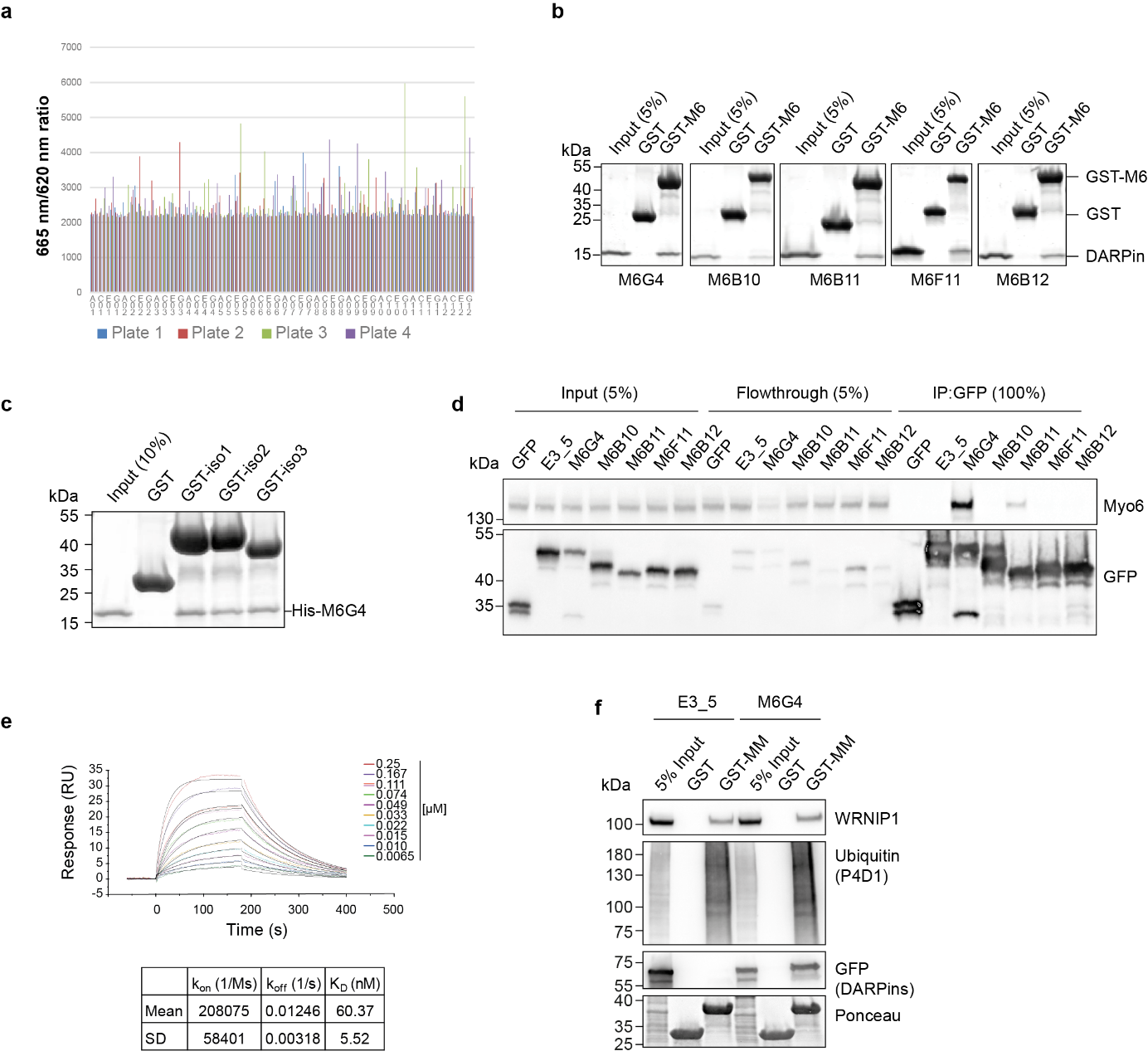
**

**Supplementary Fig. 4 Selection and characterization of the myosin VI-specific DARPin M6G4**

**a**, HTRF screening of bacterial crude extracts of expressed DARPin clones. 380 single DARPin clones encoded in the expression plasmid pQIq were picked from agar selection plates and expression was induced in four 96-well deep well plates (plate 1 to 4). After detergent lysis using B-PER (Pierce), HTRF was performed to detect binding of the FLAG-tagged DARPins to the biotinylated target myosin VI isoform 1. The ratio of readings at 665 nm divided by reading at 620 nm multiplied by 10,000 reflects the degree of specific binding. x-axis: position of the well of the 96 well expression plate.

**b**, DARPin screening via GST-pulldown assay. GST-pulldown assay using GST or GST-MyUb (labeled GST-M6) as bait and purified DARPin candidates as prey. Proteins were separated via SDS-PAGE and stained using Instant Blue.

**c**, M6G4 is a pan-isoform-specific binder. GST-pulldown assay was performed using GST or the indicated GST-MIUMyUb isoforms as bait and purified His-M6G4 as prey. Proteins were separated via SDS-PAGE and stained using Instant Blue.

**d**, DARPin M6G4 depletes myosin VI from cellular lysates. HeLa cells were transfected with the indicated GFP-tagged DARPins for 24 h. Immunoprecipitations (IPs) against GFP using GFP-trap beads (Chromotek) were performed, followed by western blotting with antibodies against myosin VI and GFP.

**e**, M6G4 binds the MIUMyUb domains with a KD of 60 nM. Surface Plasmon Resonance measurements were performed on a Biacore X100 instrument. Mean values and standard deviation (SD) from four experiments are listed below a graph showing one representative assay.

**f**, M6G4 does not compete with WRNIP1- and ubiquitin-binding to myosin VI. HEK293 cells were transfected with GFP-tagged DARPin constructs as indicated for 24 h. Cells were lysed and GST-pulldown assays were performed using GST or GST-MIUMyUb as bait. Proteins were separated via SDS-PAGE and analyzed by western blotting using the indicated antibodies. Ponceau S staining was performed to control for equal loading.

For panels **b-f:** Results were confirmed by at least two independent experiments. Uncropped blots are shown at the end of this file.

**
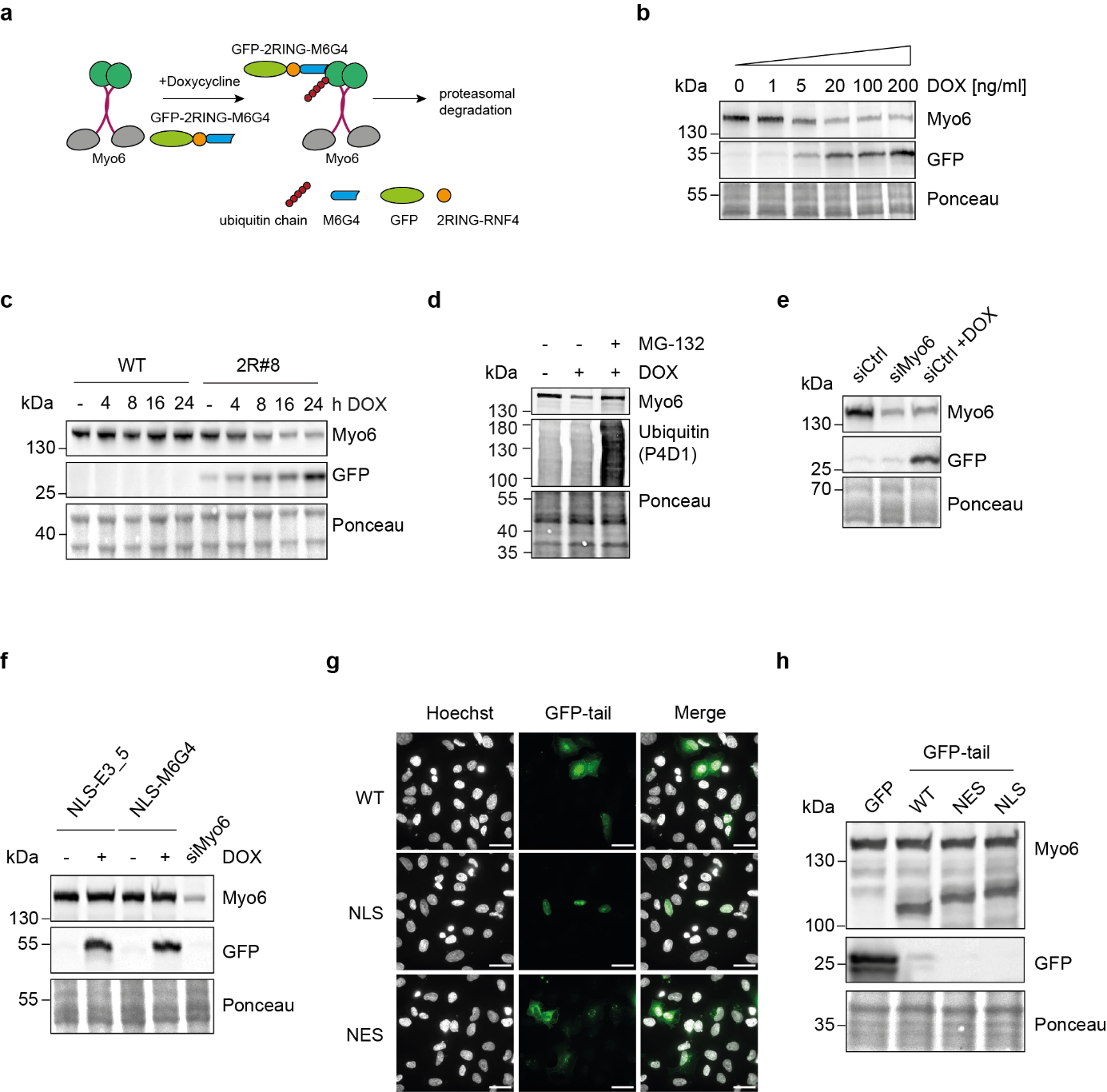
**

**Supplementary Fig. 5 Establishment of DARPin-based tools and dominant-negative constructs to study contributions of cytoplasmic versus nuclear pools of myosin VI**

**a**, Schematic representation of the DARPin-based myosin VI-degradation system adapted from Ibrahim et al.[^1^](#_ENREF_1).

**b**,**c**, A U2OS Flp-In T-REx single cell clone harboring DOX-inducible GFP-M6G4-2RING fusion construct (2R#8) was treated with increasing concentrations of DOX for 24 h **(b)** or with 20 ng/ml DOX for the indicated times **(c)**, followed by western blotting using antibodies against myosin VI and GFP. Ponceau S staining was performed to control for equal loading.

**d**, The M6G5-2RING fusion targets endogenous myosin VI for proteasomal degradation. A U2OS Flp-In T-REx single-cell clone harboring a DOX-inducible GFP-M6G4-2RING fusion construct (2R#8) was treated with 2 µg/ml DOX for 24 h and 5 µM proteasome inhibitor MG-132 as indicated. Cellular lysates were analyzed via western blotting using antibodies against myosin VI and ubiquitin. Ponceau S staining was used to compare loading between samples.

**e**,**f**,**h**, Western blots to monitor knockdown efficiencies and expression levels for the fiber assays shown in Fig. **4**. Panel **e** relates to **Fig. 4c**: U2OS Flp-In T-REx cells harboring DOX-inducible GFP-M6G4-2RING fusion construct (2R#8) were transfected with siRNAs for 72 h and treated with DOX for 24 h as indicated, followed by western blotting using antibodies against myosin VI and GFP. Panel **f** relates to **Fig. 4e**: U2OS Flp-In T-REx cells harboring DOX-inducible GFP-M6G4-NLS or GFP-E3_5-NLS (control) fusion constructs were transfected with siRNAs against myosin VI and treated with DOX as indicated, followed by western blotting using antibodies against myosin VI and GFP. Panel **h** relates to **Fig. 4f**: U2OS cells were transfected with GFP-tail constructs for 24 h as indicated, followed by western blotting using antibodies against myosin VI and GFP. Ponceau S staining was performed to control for equal loading.

**g**, Compartment-specific localization of tagged myosin VI-tails. U2OS cells were transfected with GFP or GFP-tail constructs as indicated. Nuclei were stained with Hoechst (white signal) (scale bar = 40 µm).

For panels **b-h:** Results were confirmed by at least two independent experiments. Uncropped blots are shown at the end of this file.


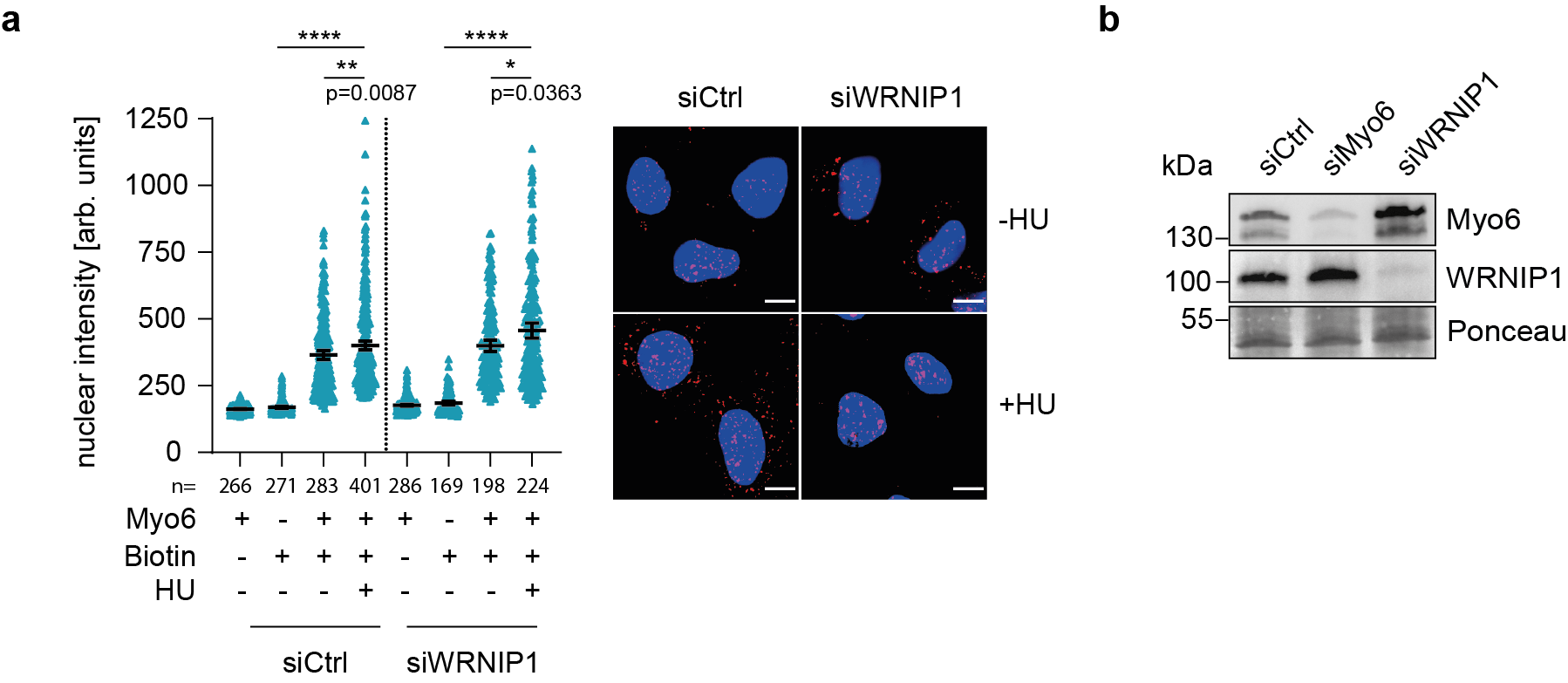


**Supplementary Fig. 6 WRNIP1 depletion has no effect on the association of myosin VI with replication forks**

**a**, U2OS cells were transfected with siRNAs for 72 h as indicated, followed by SIRF assay. Left: dot plots of PLA signal intensities with mean values -/+ 95 % confidence interval. Significance levels were calculated using the two-tailed Mann-Whitney test from the indicated number of nuclei per sample (ns, non-significant, ****: p<0.0001, ***: p<0.001, *: p<0.05). Right: representative images with Hoechst staining in blue and PLA signals in magenta, scale bar = 10 µm.

**b**, Western blot analysis to monitor knockdown efficiencies for SIRF assays shown in Fig. **5c** and panel **a**. Uncropped blots are shown at the end of this file.

For panels **a-b**: Results were confirmed by at least two independent experiments.

**Nucleotide sequence of the lead DARPin M6G4**

GACCTGGGTAAGAAACTGCTGGAAGCTGCTTGGTACGGTCAGGACGACGAAGTTCGTATCCTGATGGCAAACGGTGCTGACGTTAACGCTACTGACGACTACGGTGCTACTCCGCTGCACCTGGCTGCTCATCAGGGTCACCTGGAAATCGTTGAAGTTCTGCTGAAGGCCGGCGCTGACGTTAACGCTGACGACCAGATGGGTTTCACTCCGCTGCACCTGGCTGCTTACCATGGTCACCTGGAAATCGTTGAAGTTCTGCTGAAAACCGGTGCTGACGTTAACGCTGAAGACATCGTTGGTTTCACTCCGCTGCACCTGGCTGCTTGGTTCGGTCACCTGGAAATCGTTGAAGTTCTGCTGAAGCACGGCGCCGACGTTAACGCTCAGGACATCGTTGGTAAAACTCCGTTCGACCTGGCTATCGACAACGGTAACGAGGACATCGCTGAAGTTCTGCAGAAATCTGCTAAG

**Amino acid sequence of the lead DARPin M6G4**

| N-cap | 1st repeat |
| --- | --- |
| DLGKKLLEAAWYGQDDEVRILMANGADVNA | TDDYGATPLHLAAHQGHLEIVEVLLKAGADVNA |

| 2nd repeat | 3rd repeat |
| --- | --- |
| DDQMGFTPLHLAAYHGHLEIVEVLLKTGADVN | AEDIVGFTPLHLAAWFGHLEIVEVLLKHGADVNA |

| C-cap |
| --- |
| QDIVGKTPFDLAIDNGNEDIAEVLQKSAK |

**Supplementary Fig. 7 Nucleotide and amino acid sequence of the myosin VI-specific DARPin M6G4**

**Supplementary Tables**

**Supplementary Table 1. Plasmids used in this study**

| **Plasmids** | Source | ID, Cat# |
| --- | --- | --- |
| pGEX6P1 | Merck | Cytiva 28-9546-48 |
| pGEX6P1-MyUb | He et al.[^2^](#_ENREF_2) | 2893 |
| pBirAcm | Avidity | AVB99 |
| pAC4 | Avidity | pAC4 |
| pET30-MIUMyUb-iso1-AVI-His | This study | 3392 |
| pET30-MIUMyUb-iso2-AVI-His | This study | 3393 |
| pET30-MIUMyUb-iso3-AVI-His | This study | 3394 |
| pENTR4-myo6-iso2 | This study | 4382 |
| pDEST-TO-YFP-FRT-myo6 | This study | 4478 |
| pEGFP-C1 | Clontech | 2535 |
| pEGFP-C1 E3_5 (DARPin) | This study, derived from Binz et al.[^3^](#_ENREF_3) | 4008 |
| pEGFP-C1 M6B10 (DARPin) | This study | 4007 |
| pEGFP-C1 M6G4 (DARPin) | This study | 4009 |
| pEGFP-C1 M6B11 (DARPin) | This study | 4057 |
| pEGFP-C1 M6F11 (DARPin) | This study | 4058 |
| pEGFP-C1 M6B12 (DARPin) | This study | 4059 |
| pCDNA5 FRT TO GNb-2xRING | Ibrahim et al.[^1^](#_ENREF_1) | 4758 |
| pCDNA5-FRT-TO-EGFP-M6G4-2xRING | This study | 4912 |
| pCDNA5-FRT-TO-EGFP-E3_5-2xRING | This study | 5056 |
| pENTR4-3xNLS-M6G4 | This study | 5053 |
| pDEST-FRT-TO-YFP-3xNLS-M6G4 | This study | 5055 |
| pENTR4-3xNLS-E3_5 | This study | 5052 |
| pDEST-FRT-TO-YFP-3xNLS-E3_5 | This study | 5054 |
| pOG44 –Flp-Recombinase | Thermo Fisher Scientific | V600520 |
| pEGFP-C1-myo6 (fl) | Wollscheid et al.[^4^](#_ENREF_4) | 2905 |
| pEGFP-C1-myo6-tail (aa732-1262) | Wollscheid et al.[^4^](#_ENREF_4) | 2908 |
| pEGFP-C1-myo6-tail RRL🡪AAA | This study | 5356 |
| pEGFP-C1-myo6-tail ubiquitin binding mutant (A1013G+I1072A*) | This study | 5358 |
| pEGFP-C1-myo6-tail DNA binding mutant (K1165-1167A) | This study | 5498 |
| pEGFP-C1-myo6-tail lipid binding mutant (K1093A, K1095A) | This study | 5384 |
| pEGFP-C1-myo6-tail cargo binding mutant (WLY) | This study | 5383 |
| p3xFlag-CMV-YFP-3xNLS-myo6-tail | This study | 5463 |
| p3xFlag-CMV-YFP-NES-myo6-tail | This study | 5453 |
| pENTR221-SSBP1 | Orfeome OCAA | 3183 |
| pENTR221-PolD1 | Orfeome OCAA | 2925 |
| pENTR221-PDS5A | Orfeome OCAA | 3185 |
| pENTR221-RUVBL1 | Orfeome OCAA | 3187 |
| pENTR221-RUVBL2 | Orfeome OCAA | 3188 |
| pENTR221-WRNIP1 | Orfeome OCAA | 3184 |
| pENTR221-PRMT5 | Orfeome OCAA | 3193 |
| pDEST-EGFP-SSBP1 | This study | 42340 |
| pDEST-EGFP-PolD1 | This study | 39024 |
| pDEST-EGFP-PDS5A | This study | 49948 |
| pDEST-EGFP-RUVBL1 | This study | 38500 |
| pDEST-EGFP-RUVBL2 | This study | 34352 |
| pDEST-EGFP-WRNIP1 | This study | 5313 |
| pDEST-EGFP-PRMT5 | This study | 37484 |
| pcDNA6.2 N-EmGFP-UBR5-V5 | Addgene (#52050) | 2689 |
| pET15b-TEVs-F-USP2cat-A | This study | 3239 |
| pET3a-Ubi(K63R) | Parker et al.[^5^](#_ENREF_5) | 684 |
| pET21(+)-His6-Ubc13 | Renz et al.[^6^](#_ENREF_6) | 3010 |
| pET30-His6-TEV-Mms2 | Renz et al.[^6^](#_ENREF_6) | 2807 |
| pET30-His6-TEV-Pib1RING+100aa | Renz et al.[^6^](#_ENREF_6) | 3019 |
| pET28-mE1 (ubiquitin activating enzyme E1) | Carvalho et al.[^7^](#_ENREF_7) | 2448 |

*: I1072 in this construct (isoform 2) corresponds to I1104 in the long isoform (3). Mutation to alanine abrogates ubiquitin binding of the MyUb domain.

**Supplementary Table 2. Antibodies used in this study**

| **Antibody** | **Source** | **Identifier** |
| --- | --- | --- |
| Mouse monoclonal anti-GFP (clone 7.1/13.1) | Roche | Cat#11814460001;  RRID: AB_390913 |
| Mouse monoclonal anti- Myosin VI (clone MUD19) | Merck | Cat#M0691 RRID:AB_369989 |
| Rabbit polyclonal anti-Myosin VI | Wollscheid et al.[^4^](#_ENREF_4)  (obtained from Simona Polo, IFOM in Milan, Italy) | n/a |
| Rabbit polyclonal anti- WRNIP1 | Bethyl laboratories | Cat#A301-389A-T  RRID:AB_938087 |
| Mouse monoclonal anti-WHIP (clone A-8) | Santa Cruz Biotechnology | Cat#sc-376438  RRID:AB_11149006 |
| Rabbit polyclonal anti-DNA-PKcs | Cell Signaling Technology | Cat#4602  RRID:AB_10692482 |
| Rabbit polyclonal anti-53BP1 | Novus Biologicals | Cat#NB100-304  RRID:AB_10003037 |
| Rabbit polyclonal anti-SMC1 | Bethyl Laboratories | Cat#A300-055A  RRID:AB_66638 |
| Rabbit monoclonal anti-VCP (clone 7F3) | Cell Signaling Technology | Cat#2649  RRID:AB_2214629 |
| Rabbit polyclonal anti-MCM7 (clone D10A11) | Cell Signaling Technology | Cat#3735  RRID:AB_2142705 |
| Mouse monoclonal anti-Rad18 (clone 79B1048) | Abcam | Cat#ab12007  RRID:AB_298783 |
| Rabbit monoclonal anti-Rad21 (clone D5Y8S) | Cell Signaling Technology | Cat#12673  RRID:AB_2797988 |
| Mouse polyclonal anti-RFC4 | Abcam | Cat#ab2627  RRID:AB_303218 |
| Mouse monoclonal anti-PCNA (clone PC10) | David P. Lane[^8^](#_ENREF_8)  (obtained from Bořivoj Vojtěšek, Masaryk Memorial Cancer Institute in Brno, Czech Republic) | n/a |
| Rabbit polyclonal anti-PCNA | Abcam | Cat#ab18197  RRID:AB_444313 |
| Mouse monoclonal anti-BRCA2 (clone 2B) | Merck | Cat#OP95  RRID:AB_213443 |
| Rabbit monoclonal anti-Rad51 (clone D4B10) | Cell Signaling Technology | Cat#8875  RRID:AB_2721109 |
| Rabbit polyclonal anti-HLTF (clone ART2) | Alexandra Belayew[^9^](#_ENREF_9)  (obtained from Alexandra Belayew , UMONS in Mons, Belgium) | n/a |
| Mouse monoclonal anti-SMARCAL1 (clone A-2) | Santa Cruz Biotechnology | Cat#sc-376377  RRID:AB_10987841 |
| Rabbit polyclonal anti-ZRANB3 | Bethyl Laboratories | Cat#A303-033A |
| Mouse monoclonal anti-Biotin (clone Hyb-8) | Abcam | Cat#ab201341  RRID:AB_2861249 |
| Mouse monoclonal anti-BrdU (clone B44) (IdU) | BD Biosciences | Cat#347580  RRID:AB_400326 |
| Rat monoclonal anti-BrdU (clone BU1/75 (ICR1) ) (CldU) | Abcam | Cat#ab6326  RRID:AB_305426 |
| Rabbit polyclonal anti-phospho-Chk1 (Ser345) | Cell Signaling Technology | Cat#2341  RRID:AB_330023 |
| Mouse monoclonal anti-Chk1 (clone 2G1D5) | Cell Signaling Technology | Cat#2360  RRID:AB_2080320 |
| Rabbit polyclonal anti-phospho-Chk 1 (Ser317) | Cell Signaling Technology | Cat#2344  RRID:AB_331488 |
| Mouse monoclonal anti-polyHistidine (clone HIS-1) | Merck | Cat#H1029  RRID:AB_260015 |
| Mouse monoclonal anti-β-actin (clone AC-74) | Merck | Cat#A2228  RRID:AB_476697 |
| Mouse monoclonal anti-ubiquitin (clone P4D1) | Cell Signaling Technology | Cat#3936  RRID:AB_331292 |
| IRDye® 680LT donkey anti-rabbit IgG secondary  Antibody | LICOR | Cat#926-68023;  RRID: AB_10706167 |
| IRDye® 680LT donkey anti-mouse IgG secondary  antibody | LICOR | Cat#926-68072;  RRID: AB_10953628 |
| IRDye® 800CW goat anti-rabbit IgG secondary antibody | LICOR | Cat#926-32211;  RRID: AB_621843 |
| IRDye® 800CW donkey anti-mouse IgG secondary antibody | LICOR | Cat#926-32212;  RRID: AB_621847 |
| Polyclonal goat anti-goat HRP secondary antibody | Dako | Cat# P044901-2  RRID:AB_2617143 |
| Polyclonal goat anti-mouse HRP secondary antibody | Dako | Cat#P044701-2  RRID:AB_2617137 |
| Polyclonal goat anti-rabbit HRP secondary antibody | Dako | Cat#P044801-2  RRID:AB_2617138 |
| Goat anti-Rat IgG (H+L) Secondary Antibody, Alexa Fluor 488 | Thermo Fisher Scientific | Cat#A-11006  RRID:AB_2534074 |
| Goat anti-Mouse IgG (H+L) Secondary Antibody, Alexa Fluor 647 | Thermo Fisher Scientific | Cat#A-21236  RRID:AB_2535805 |
| Goat anti-Rabbit IgG (H+L) Cross-Adsorbed, Alexa Fluor 647 | Thermo Fisher Scientific | Cat#A-21244  RRID:AB_2535812 |

**Supplementary Table 3. siRNAs used in this study**

| **siRNA** | **Source** | **Identifier** |
| --- | --- | --- |
| Allstars Negative control siRNA | Qiagen | Cat#SI03650318 |
| Hs_MYO6_5 FlexiTube siRNA | Qiagen | Cat#SI03142692 |
| Hs_MYO6_7 FlexiTube siRNA | Qiagen | Cat#SI04243351 |
| Hs_MYO6_8 FlexiTube siRNA | Qiagen | Cat#SI04370737 |
| Hs_MYO6_10 FlexiTube siRNA | Qiagen | Cat#SI04998749 |
| FlexiTube GeneSolution GS56897 for WRNIP1 | Qiagen | Cat# GS56897 |
| FlexiTube GeneSolution GS675 for BRCA2 | Qiagen | Cat#GS675 |
| SiRNA Silencer Select [Hs] RAD51 | Thermo Fisher Scientific | Cat#s531930 |
| SiRNA Silencer Select [Hs] RAD51 | Thermo Fisher Scientific | Cat#s11734 |
| siRNA Silencer Select [Hs] HLTF | Thermo Fisher Scientific | Cat#s13137 |
| SiRNA Silencer Select [Hs] ZRANB3 | Thermo Fisher Scientific | Cat#s38488 |
| SiRNA Silencer Select [Hs] ZRANB3 | Thermo Fisher Scientific | Cat#s224929 |
| Hs_SMARCAL1_3 FlexiTube siRNA | Qiagen | Cat#SI00103194 |
| Hs_SMARCAL1_1 FlexiTube siRNA | Qiagen | Cat#SI00103180 |

**Supplementary Table 4. Oligonucleotides used in this study**

| **Oligonucleotides for mutagenesis** | Sequence (5´- 3´) | Construct | ID |
| --- | --- | --- | --- |
| Myo6 RRL🡪 AAA For | GGCAGCTTGCAGAGAAGAATTTCATGCGGCAGCAAAAGTGTATCATGCTTGGAAATCTAAG | pEGFP-C1-myo6-tail RRL🡪AAA | 4898 |
| Myo6 RRL🡪 AAA Rev | CTTAGATTTCCAAGCATGATACACTTTTGCTGCCGCATGAAATTCTTCTCTGCAAGCTGCC |  | 4899 |
| Myo6 A1013G For | CGGCTTCACTCTGGGCAATCCTCAGGCCCAGCTCCCGGTCCCTGCGCTCC | pEGFP-C1-myo6-tail ubiquitin binding mutant (A1013G+I1072A) | 4896 |
| Myo6 A1013G Rev | CGGCTTCACTCTGGGCAATCCTCAGGCCCAGCTCCCGGTCCCTGCGCTCC |  | 4897 |
| Myo6 I1072A* For | CGTGATACCATCAATACTTCTTGTGATGCTGAGCTCCTGGCAGCTTGCAGAGAAGAATTTCATAGG |  | 5250 |
| Myo6 I1072A* Rev | CCTATGAAATTCTTCTCTGCAAGCTGCCAGGAGCTCAGCATCACAAGAAGTATTGATGGTATCACG |  | 5251 |
| Myo6 DNA bdg mut For | GCCGACCAGTACAAAGACCCTCAGGCTGCGGCAGCAGGCTGGTGGTATGCCC | pEGFP-C1-myo6-tail DNA binding mutant (K1165-1167A) | 6223 |
| Myo6 DNA bdg mut Rev | GGGCATACCACCAGCCTGCTGCCGCAGCCTGAGGGTCTTTGTACTGGTCGGC |  | 6224 |
| Myo6 lipid bdg mut For | GGAGACTAAAAGTGTATCATGCTTGGGCATCTGCGAACAAGAAGAGAAATACTGAAACAGAGCAACGTGCTCC | pEGFP-C1-myo6-tail lipid binding mutant (K1093A, K1095A) | 6068 |
| Myo6 lipid bdg mut Rev | GGAGCACGTTGCTCTGTTTCAGTATTTCTCTTCTTGTTCGCAGATGCCCAAGCATGATACACTTTTAGTCTCC |  | 6069 |
| Myo6 WWY🡪WLY For | CCCTCAGAGTAAGAAAAAAGGCTGGTTGTATGCCCATTTTGATGGACCATGGATTGCCCGG | pEGFP-C1-myo6-tail cargo binding mutant (WLY) | 6064 |
| Myo6 WWY🡪WLY Rev | CCGGGCAATCCATGGTCCATCAAAATGGGCATACAACCAGCCTTTTTTCTTACTCTGAGGG |  | 6065 |
| **Oligonucleotide for amplification** | Sequence (5´- 3´) | Construct | ID |
| MIU FOR BamHI | CGGGATCCCAACAGCAAGCAGTTCTGGAGC | pET30-MIUMyUb-iso2-AVI-His | 3837 |
| MyUb Rev no STOP EcoRI | GGAATTCTCTCTTCTTGTTCTTAGATTTCCAAGCATG |  | 3838 |
| Myo6 KpnI FWD | GGGGTACCATGGAGGATGGAAAGCCCGTTTGGG | pENTR4-myo6-iso2 | 5055 |
| Myo6 Xba REV | GCTCTAGACTACTTTAACAGACTCTGCAGCATGGC |  | 5056 |
| EGFP for_HINDIII | CCCAAGCTTATGGTGAGCAAGGGCGAGGA | pCDNA5-FRT-TO-EGFP-M6G4-2xRING | 5597 |
| M6G4 rev_NHEI | CTAGCTAGCTAGCAGATTTCTGCAGAACTTCAGCG |  | 5594 |
| E3_5_rev_NHEI | CTAGCTAGCTTGCAGGATTTCAGCCAGGTC | pCDNA5-FRT-TO-EGFP-E3_5-2xRING | 5688 |
| 3xNLS for_KpnI | GGGGTACCCCATGGGGGCCCAG | pENTR4-NLS-G4 | 5641 |
| M6G4 rev_XhoI | CCGCTCGAGTAGCAGATTTCTGCAGAACTTCAGCG |  | 5640 |
| E3_5 XhoI REV | CGGCTCGAGTTGCAGGATTTCAGCCAGGTCCTCG | pENTR4-NLS-E3_5 | 5360 |
| GFP For KpnI | GATCGGTACCGATGGTGAGCAAGGGCG | p3xFlag-CMV-YFP-NES-myo6-tail | 6189 |
| Myo6 Xba REV | GCTCTAGACTACTTTAACAGACTCTGCAGCATGGC |  | 5056 |

*: I1072 in this construct (isoform 2) corresponds to I1104 in the long isoform (3).

**Supplementary Table 5. Reagents used in this study**

| **Chemical** | **Source** | **Identifier** |
| --- | --- | --- |
| Doxycycline hydrochloride | Merck | Cat#D3447 |
| 5-Chloro-2′-deoxyuridine | Merck | Cat#C6891 |
| 5-iodo-2′-desoxyuridin | Merck | Cat#I7125 |
| 5-ethynyl-2′-deoxyuridine | Merck | Cat#900584 |
| Azide-PEG3-biotin conjugate | Merck | Cat#762024 |
| Mirin | Merck | Cat#M9948 |
| DNA2 inhibitor C5 | AOBIOUS | Cat#AOB9082 |
| IPTG | Generon | Cat#GEN-S-02122 |
| Ni-NTA agarose | Qiagen | Cat#30250 |
| Imidazole | Merck | Cat#I2399 |
| Glutathione | Merck | Cat#G4251 |
| IPTG | Generon | Cat#GEN-S-02122 |
| Paraformaldehyde | Merck | Cat#P6148 |
| Formaldehyde solution, 36.5-38% in H2O | Merck | Cat#F8775 |
| Glycine | Merck | Cat#G7126 |
| Methanol | Thermo Fisher Scientific | Cat#15654570 |
| Acetic acid | Merck | Cat#A6283 |
| SIGMAFAST protease inhibitor cocktail | Merck | Cat#S8830 |
| cOmplete Protease Inhibitor Cocktail | Roche | Cat#5056489001 |
| Hygromycin B Gold | Invivogen | Cat#ant-hg-1 |
| Pfu Turbo DNA Polymerase | Agilent | Cat#600250 |
| DpnI | New England Biolabs | Cat#R0176L |
| Hydroxyurea | Merck | Cat#H8627 |
| Streptavidin Agarose Resin | Thermo Fisher Scientific | Cat#11846764 |
| Polyethyleneimine | Polysciences | Cat#23966-2 |
| Lipofectamine® 2000 | Thermo Fisher Scientific | Cat#11668019 |
| Lipofectamine® RNAiMAX | Thermo Fisher Scientific | Cat#13778150 |
| FuGENE® HD Transfection Reagent | Promega | Cat#E2311 |
| ProLong™ Diamond Antifade Mountant | Thermo Fisher Scientific | Cat#15205739 |
| DMEM, high glucose, pyruvate, no glutamine | Thermo Fisher Scientific | Cat#21969035 |
| Trypsin‐EDTA (0.05%), phenol red | Thermo Fisher Scientific | Cat#25300054 |
| Penicillin‐Streptomycin (10,000 U/mL) | Thermo Fisher Scientific | Cat#15140122 |
| L‐Glutamine (200 mM) | Thermo Fisher Scientific |  |
| Blasticidin | Invivogen | Cat#ant-bl-1 |
| Triton X-100 | Merck | Cat#T9284 |
| Albumin (bovine serum albumin, BSA) | Merck | Cat#A7906 |
| Copper (II) sulfate | Merck | Cat#469130 |
| Sodium deoxycholate | Merck | Cat#D6750 |
| SDS, 20%, Sodium dodecyl sulfate | Merck | Cat#05030 |
| NuPAGE LDS Sample Buffer (4X) | Thermo Fisher Scientific | Cat#11559166 |
| GFP-Trap magnetic agarose beads | Chromotek | Cat#gtma-100 |
| Milk powder, skim milk | Merck | Cat#70166 |
| Tween-20 | Merck | Cat#P7949 |
| Amersham ECL Select Western Blotting Detection Reagent | Thermo Fisher Scientific | Cat#RPN2235 |
| Amersham ECL Prime Western Blotting Detection Reagent | Thermo Fisher Scientific | Cat#12994780 |
| Ponceau S | Merck | Cat#P3504 |
| Hoechst 33342 | Thermo Fisher Scientific | Cat#11534886 |
| Biotin | Merck | Cat#B4501 |
| PageRuler Prestained Protein Ladder | Thermo Fisher Scientific | Cat#11822124 |
| 4-15% Criterion™ TGX Stain-Free™ Protein Gel, 26 well, 15 µl | Bio-Rad Laboratories | Cat#567-8085 |
| Mini-PROTEAN TGX Stain Free Gels, 4-15%, 15-well | Bio-Rad Laboratories | Cat#456-8086 |
| SILAC light isotopes: L-Arg(0) and L-Lys(0) | Merck | Cat#A6969  Cat#L8662 |
| SILAC medium isotopes: L-Arg(6) and L-Lys(4) | Cambridge Isotope Laboratories | Cat#CLM-2265-H-1  Cat#DLM-2640-1 |
| Trypsin, MS-approved | Serva | Cat# 37286 |
| Glutathione Sepharose High Performance resin (GSH) | Cytiva | Cat#17527901 |
| Recombinant ubiquitin activating enzyme (E1) | In house protein production core facility | Carvalho et al^6^ |
| Ubiquitin from bovine erythrocytes | Merck | Cat#U6253-25MG |
| MG-132, 25 mg (Z-Leu-Leu-Leu-CHO) | Enzo Life Sciences | Cat#BML-PI102-0025 |
| Alexa Fluor™ 647 Phalloidin | Thermo Fisher Scientific | Cat#A22287 |
| µ-Slide 8 Well Chamber Slides | Ibidi | Cat#80806 |
| **Critical commercial assays** |  |  |
| Duolink® In Situ Red Starter Kit Mouse/Rabbit | Merck | Cat#DUO92101-1KT |
| InstantBlue, 1L Protein Stain | Biozol | Cat#EXP-ISB01L |
| Trans-Blot® Turbo™ RTA Midi Nitrocellulose Transfer Kit | Bio Rad laboratories | Cat#1704271 |
| Gateway® LR Clonase® II enzyme mix | Thermo Fisher Scientific | Cat#10134992 |
| Colloidal Blue staining kit | Thermo Fisher Scientific | Cat#LC6025 |
| Biotin CAPture kit | GE Healthcare | Cat#28920233 |

**Supplementary References**

1. Ibrahim, A.F.M. et al. Antibody RING-Mediated Destruction of Endogenous Proteins. *Mol Cell* **79**, 155-166 e159 (2020).

2. He, F. et al. Myosin VI Contains a Compact Structural Motif that Binds to Ubiquitin Chains. *Cell Rep* **14**, 2683-2694 (2016).

3. Binz, H.K., Stumpp, M.T., Forrer, P., Amstutz, P. & Plückthun, A. Designing repeat proteins: well-expressed, soluble and stable proteins from combinatorial libraries of consensus ankyrin repeat proteins. *J Mol Biol* **332**, 489-503 (2003).

4. Wollscheid, H.P. et al. Diverse functions of myosin VI elucidated by an isoform-specific alpha-helix domain. *Nat Struct Mol Biol* **23**, 300-308 (2016).

5. Parker, J.L. & Ulrich, H.D. Mechanistic analysis of PCNA poly-ubiquitylation by the ubiquitin protein ligases Rad18 and Rad5. *EMBO J* **28**, 3657-3666 (2009).

6. Renz, C. et al. Ubc13-Mms2 cooperates with a family of RING E3 proteins in budding yeast membrane protein sorting. *J Cell Sci* **133** (2020).

7. Carvalho, A.F. et al. High-yield expression in Escherichia coli and purification of mouse ubiquitin-activating enzyme E1. *Mol Biotechnol* **51**, 254-261 (2012).

8. Waseem, N.H. & Lane, D.P. Monoclonal antibody analysis of the proliferating cell nuclear antigen (PCNA). Structural conservation and the detection of a nucleolar form. *J Cell Sci* **96 ( Pt 1)**, 121-129 (1990).

9. Debauve, G. et al. Early expression of the Helicase-Like Transcription Factor (HLTF/SMARCA3) in an experimental model of estrogen-induced renal carcinogenesis. *Mol Cancer* **5**, 23 (2006).

**Uncropped images for Supplementary Figures**

**Supplementary Fig. 1a**


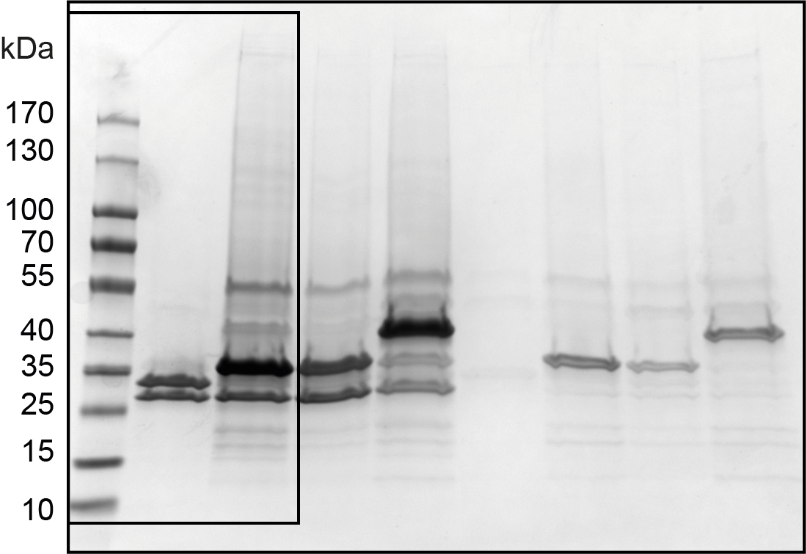


**Supplementary Fig. 1c**


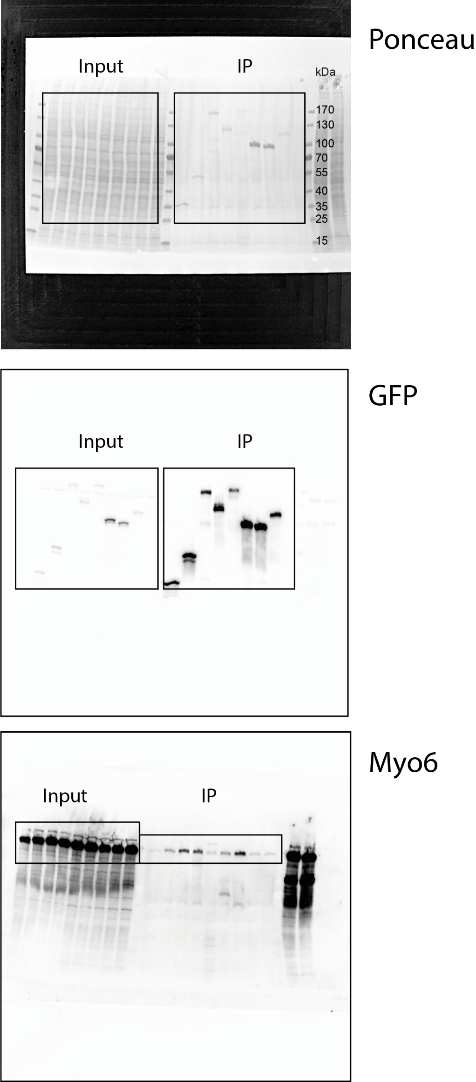


**Supplementary Fig. 2a**


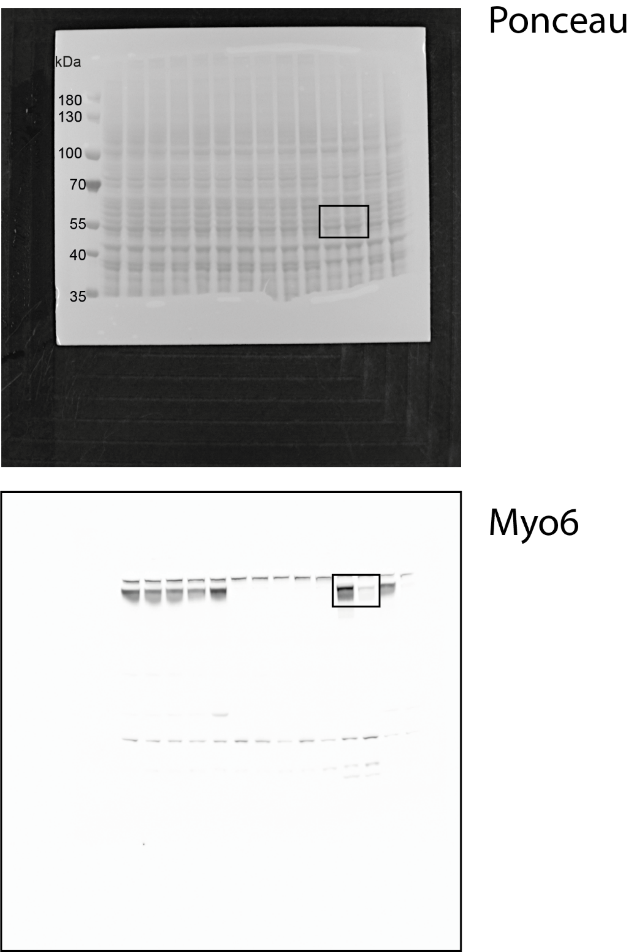


**Supplementary Fig. 2b**


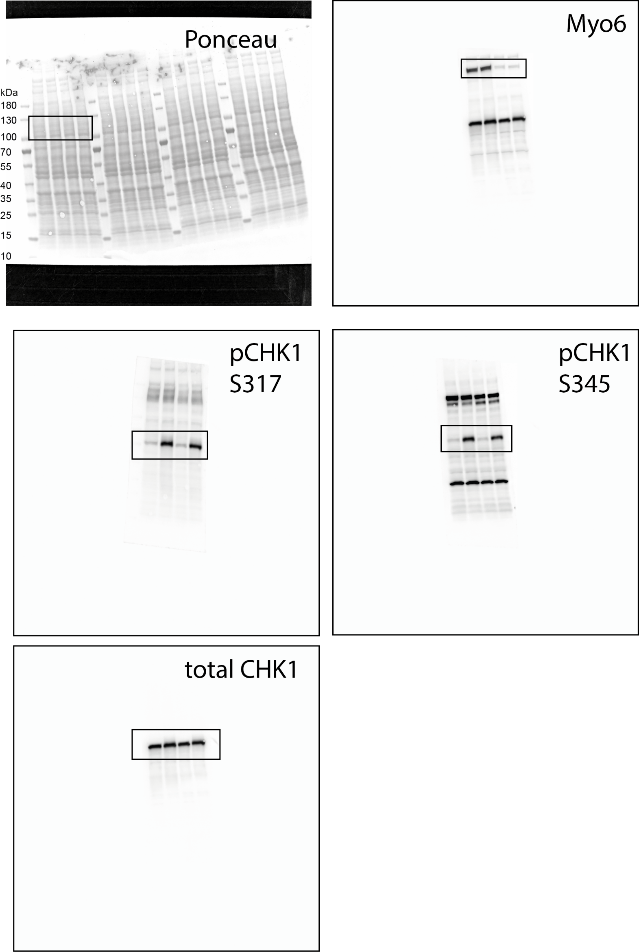


**Supplementary Fig. 2c**


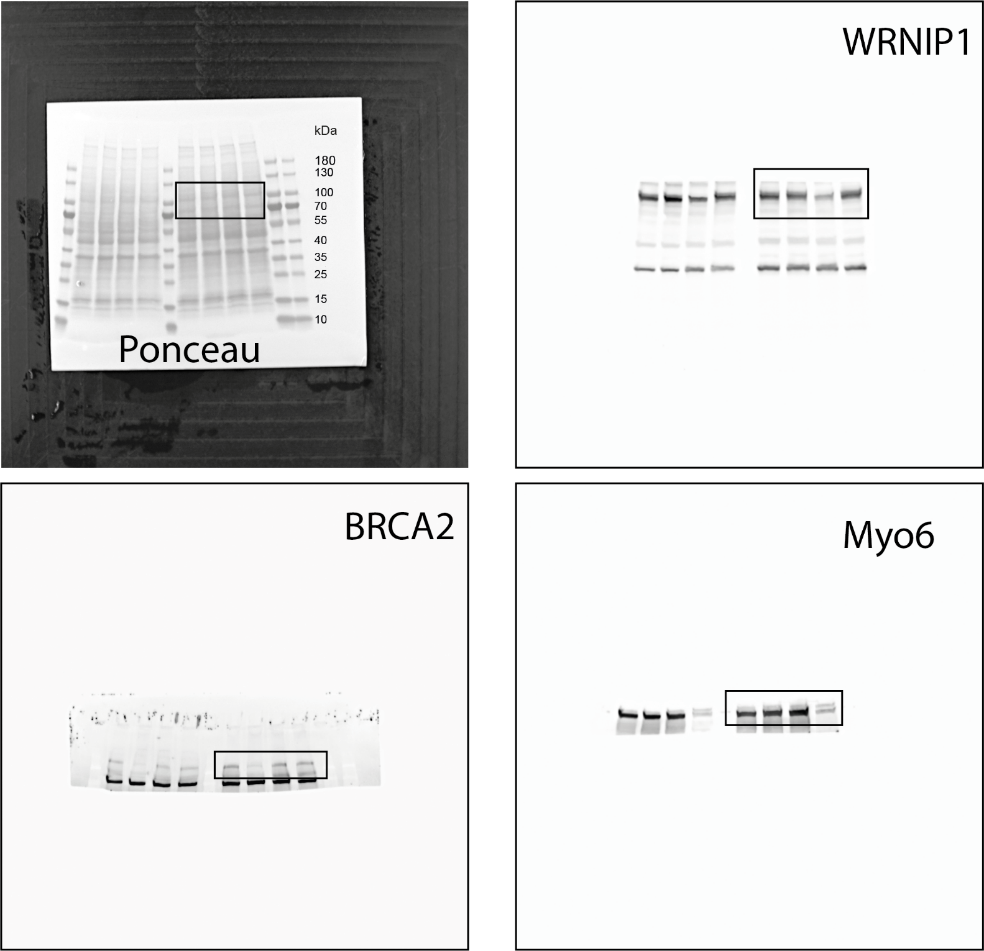


**Supplementary Fig. 2d**


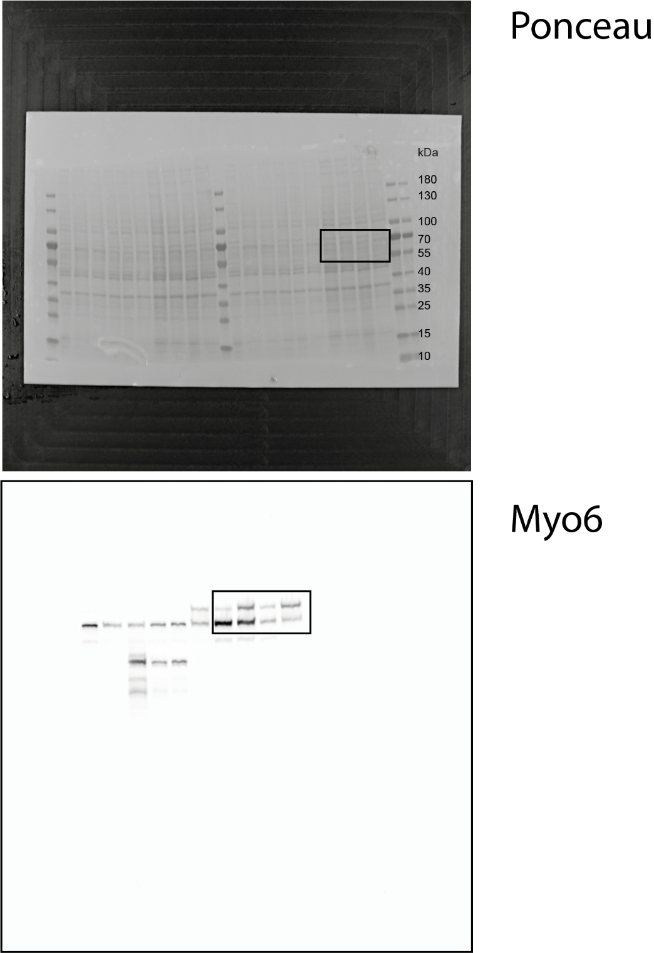


**Supplementary Fig. 2e**


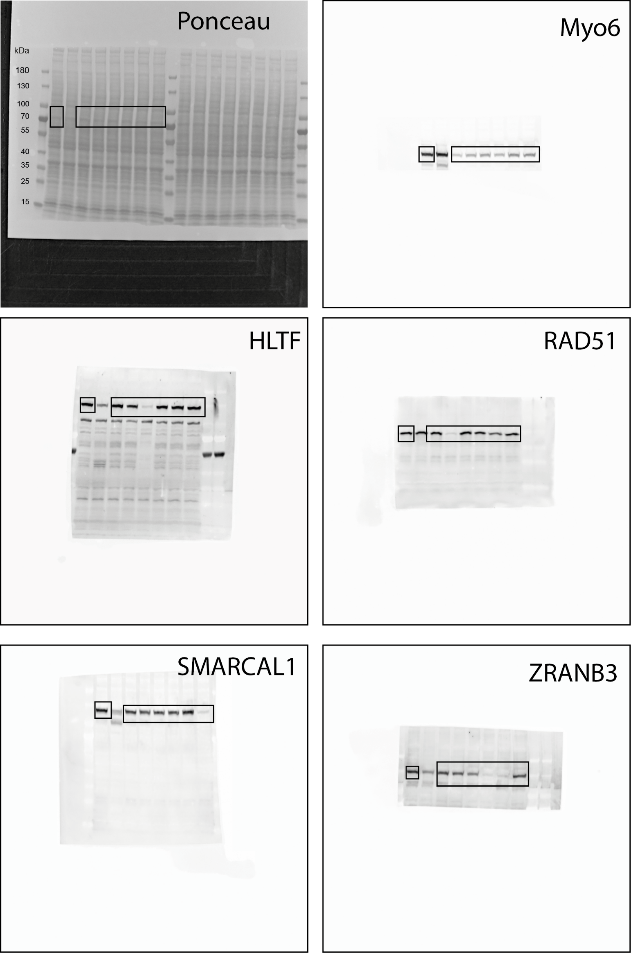


**Supplementary Fig. 2g**


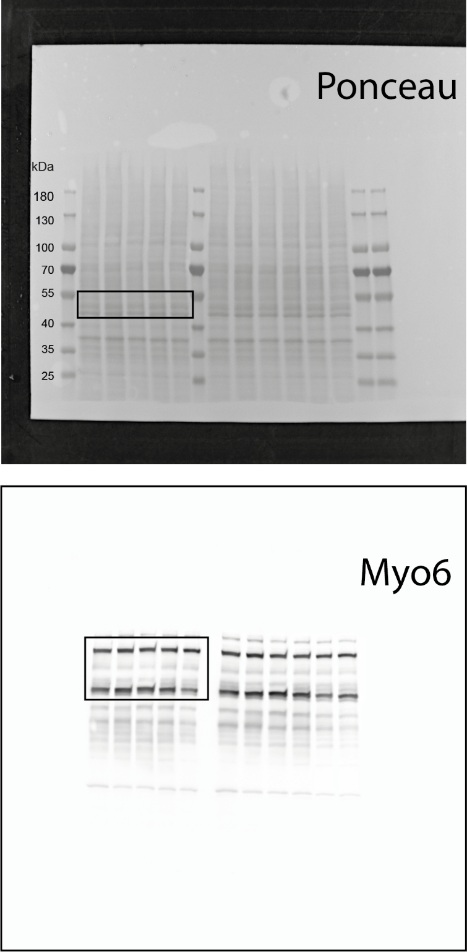


**Supplementary Fig. 3g**


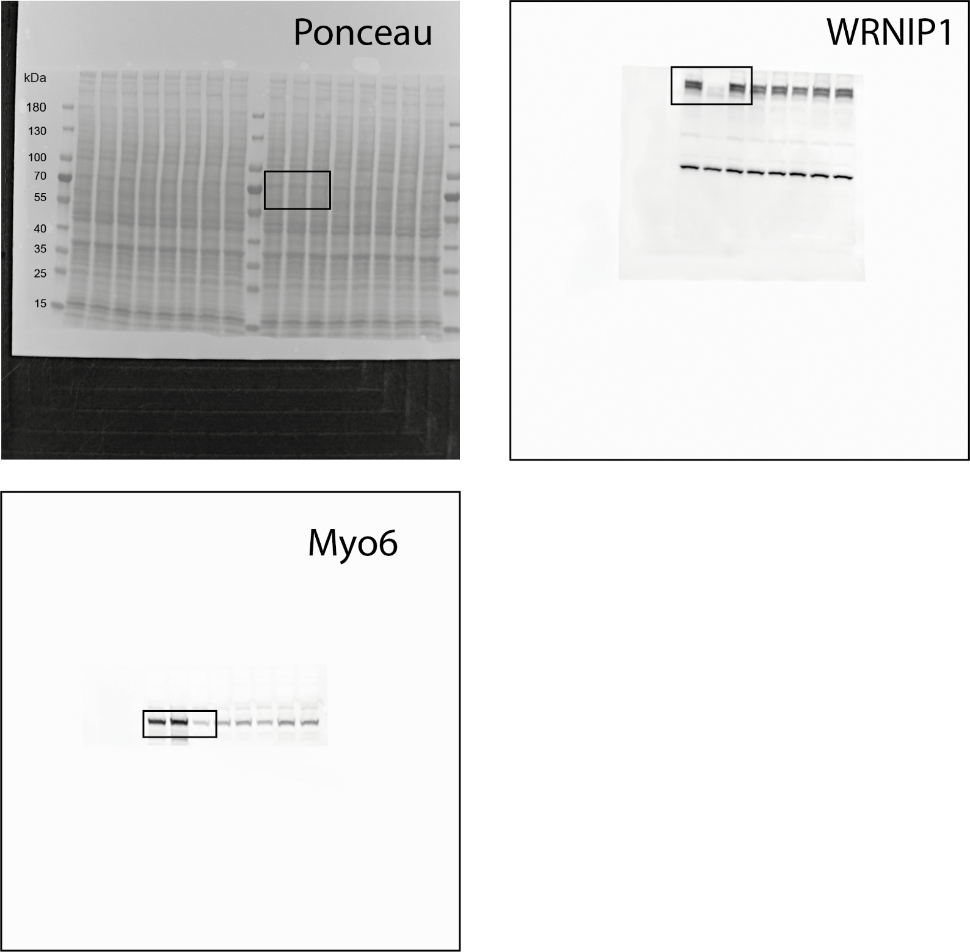


**Supplementary Fig. 4b**


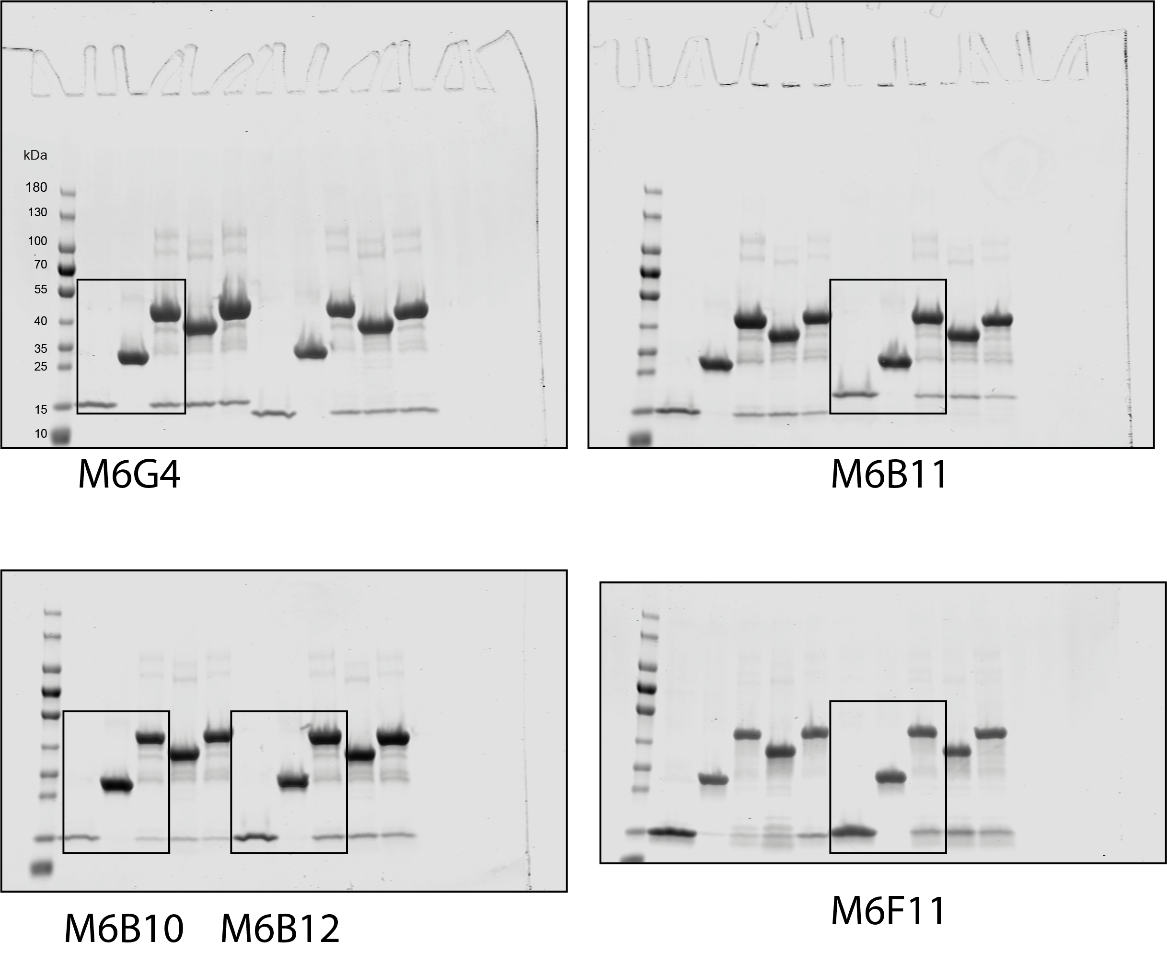


**Supplementary Fig. 4c**


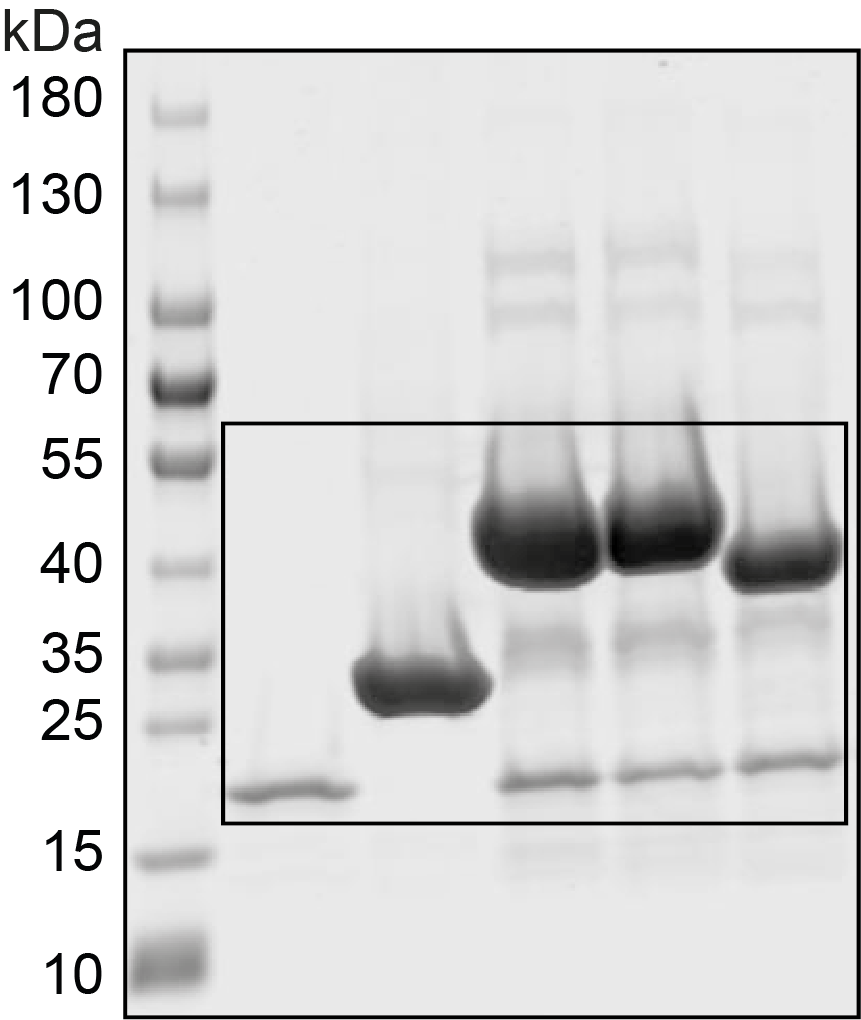


**Supplementary Fig. 4d**


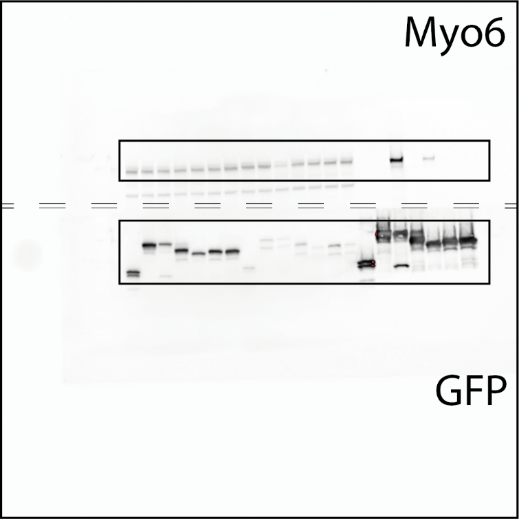


**Supplementary Fig. 4f**


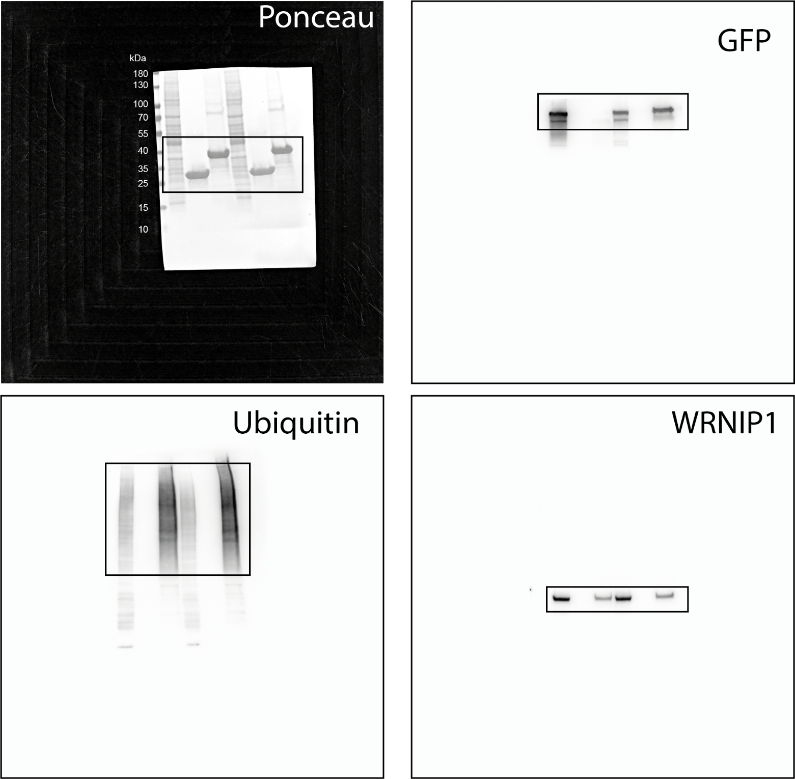


**Supplementary Fig. 5d**


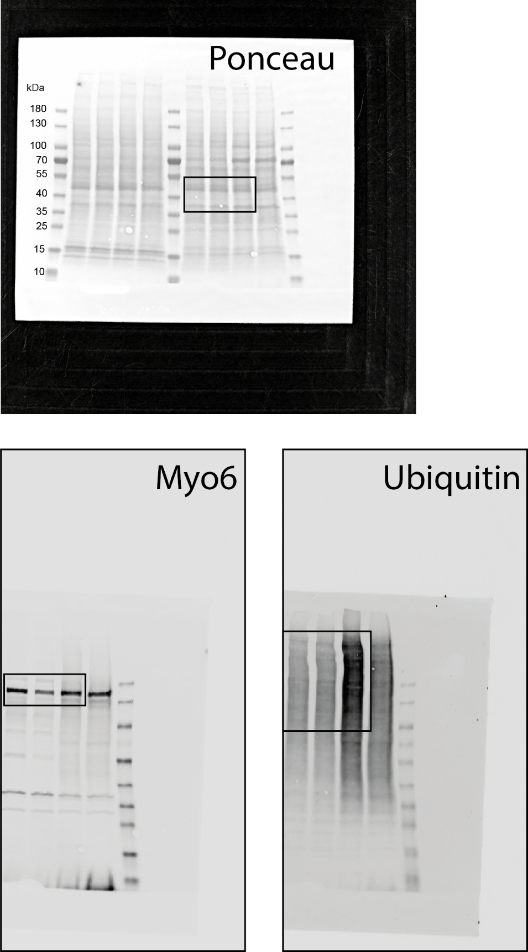


**Supplementary Fig. 5b**


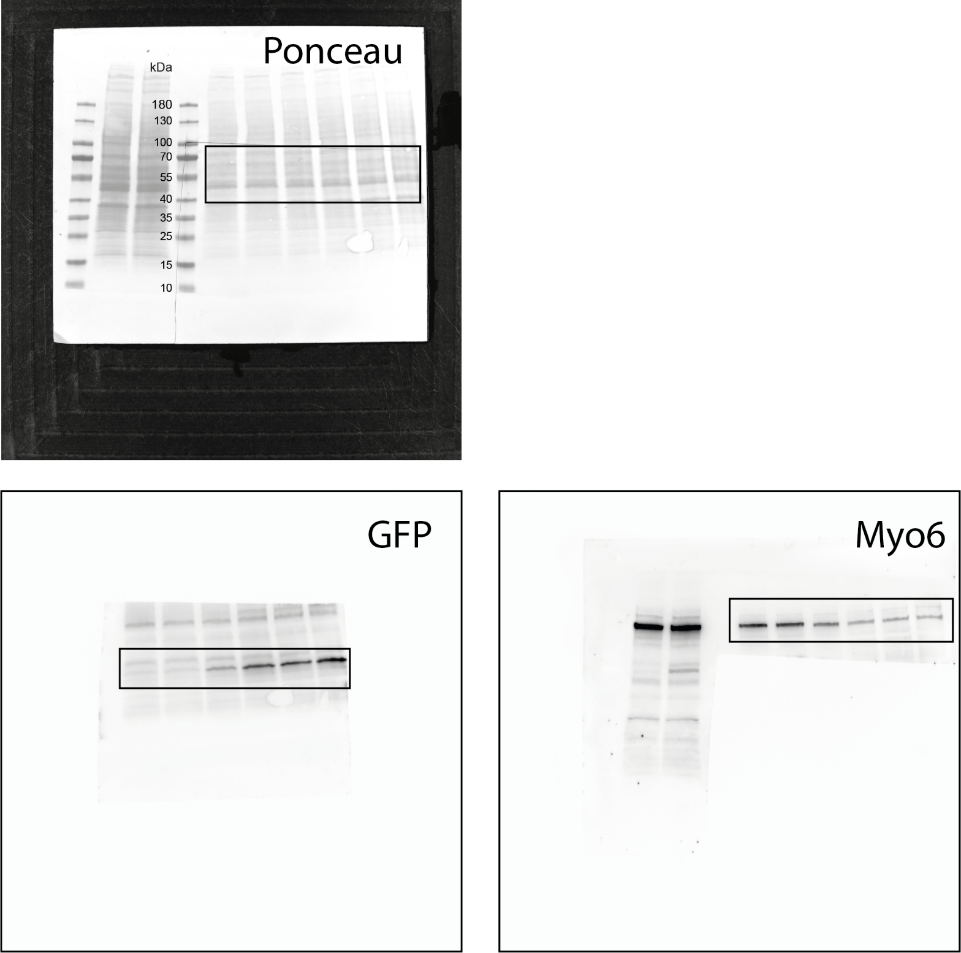


**Supplementary Fig. 5c**


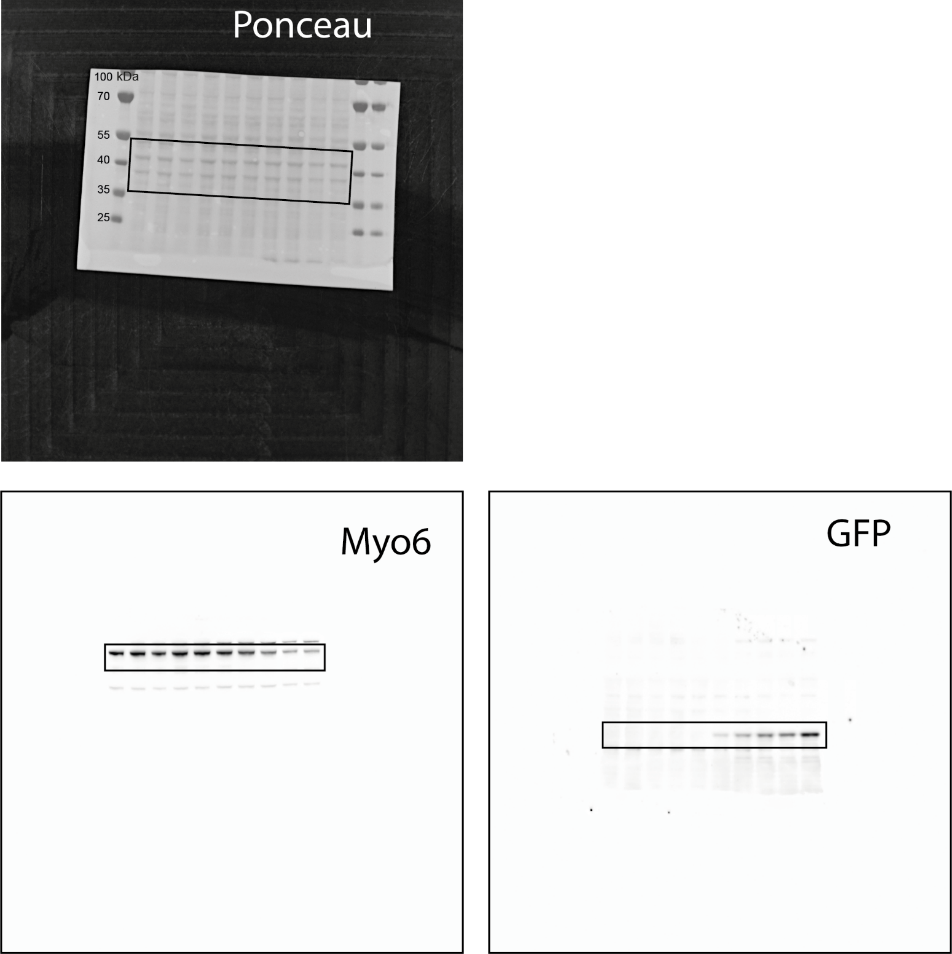


**Supplementary Fig. 5e**


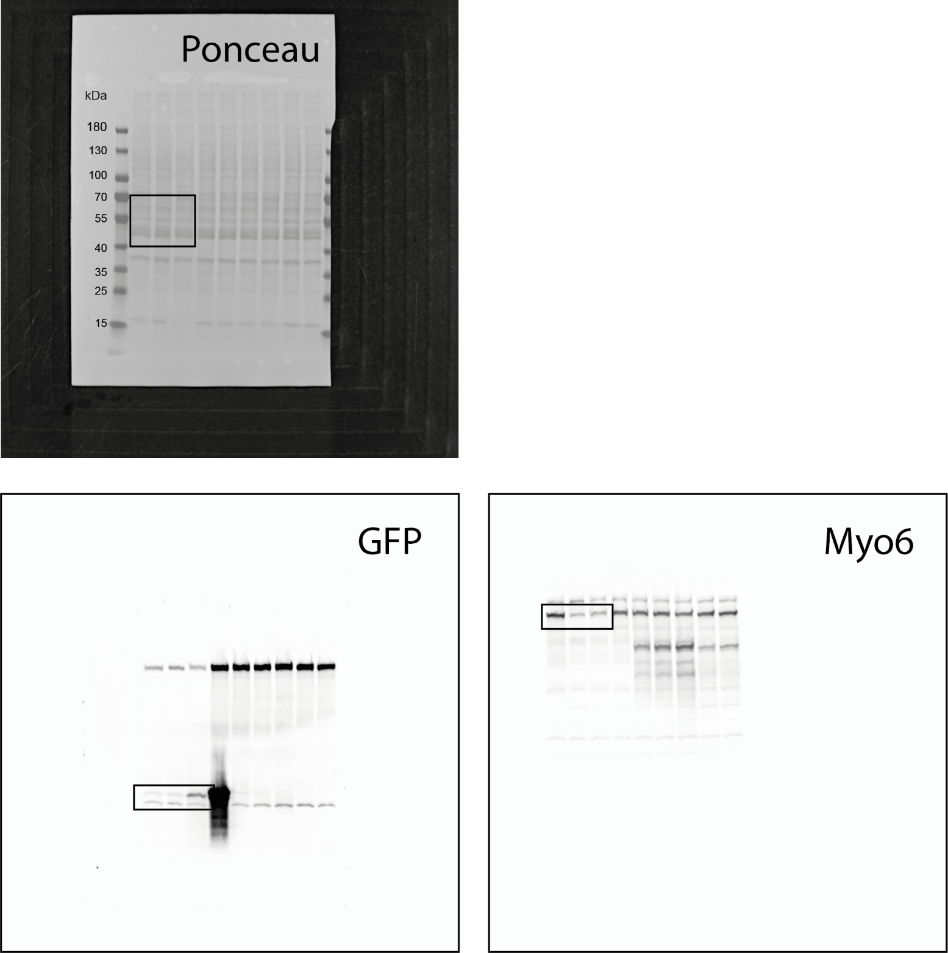


**Supplementary Fig. 5f**


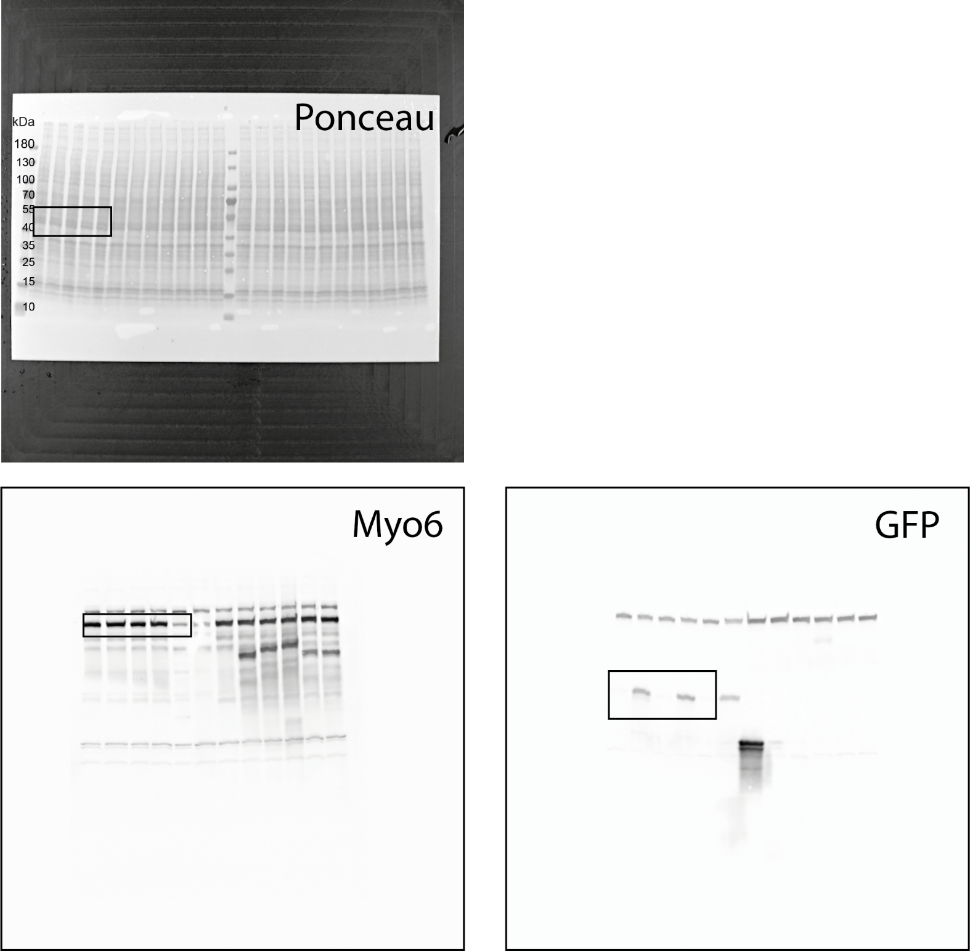


**Supplementary Fig. 5h**


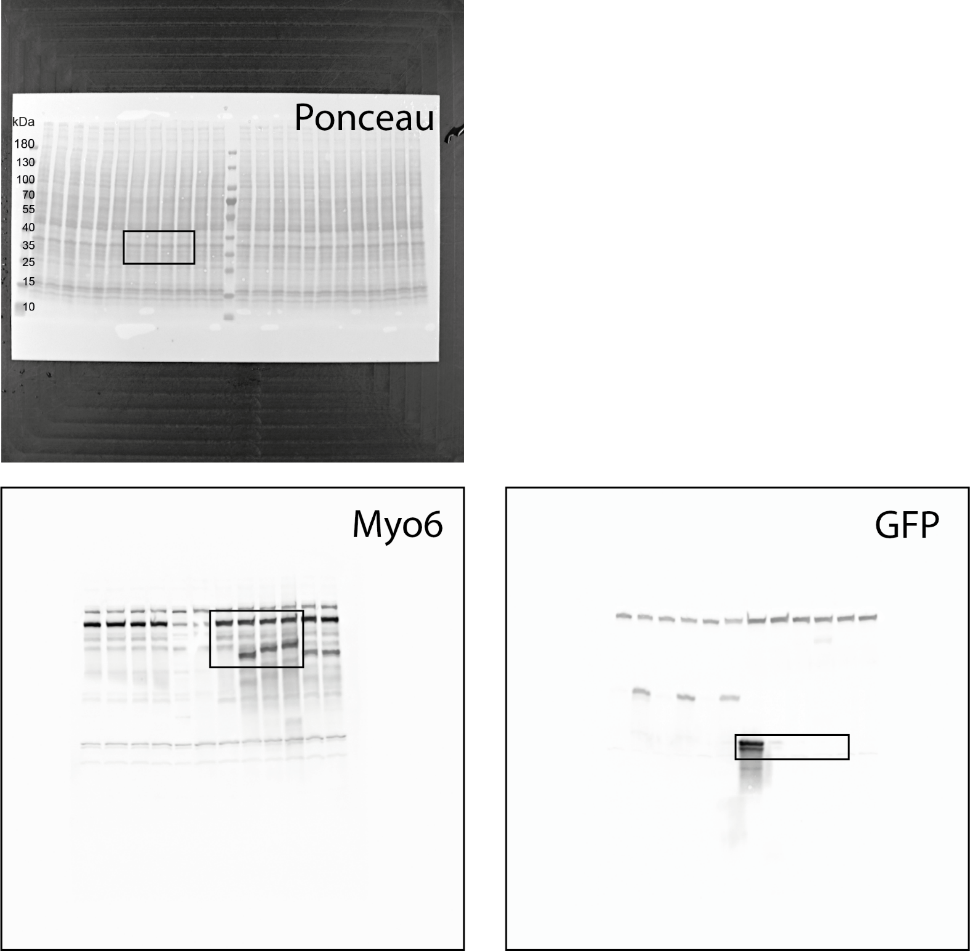


**Supplementary Fig. 6b**


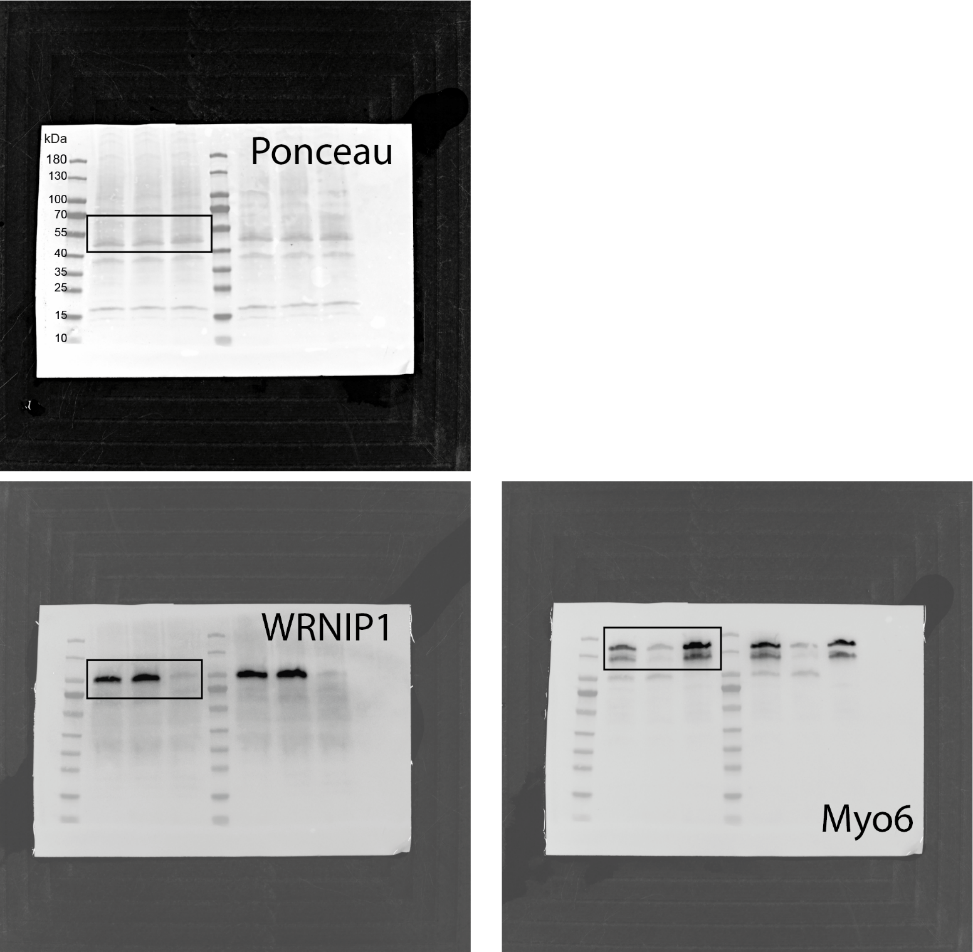

Supplement: Supplementary file 1 — Supplementary Information [file 41467_2023_39517_MOESM1_ESM.docx]
