# Supplementary material for: Nuclear myosin VI maintains replication fork stability
Source: Nat Commun. 2023 Jun 24;14:3787. doi: 10.1038/s41467-023-39517-y (PMC10290672; doi:10.1038/s41467-023-39517-y)
Supplement: Supplementary file 3 — Description of Additional Supplementary Files [file 41467_2023_39517_MOESM3_ESM.pdf]

Description of Additional Supplementary Files

File Name: Supplementary Data 1
Description: . A list of all protein groups significantly enriched in the SILAC interactome of GST-tagged MyUb (fold change >2, FDR <0.05), related to figure 1D. Statistics were derived using a two-sided moderated t-test (limma), with BH correction for multiple comparisons.

File Name: Supplementary Data 2
Description: A list of all GO cellular components mapped to proteins from Supplementary Data 1.

File Name: Supplementary Data 3
Description: A list of all GO biological processes mapped to top interactors of GST-tagged MyUb (fold change >4, FDR <0.05), related to Figure 1C. EnrichR statistics were calculated using Fisher's exact test, with BH correction for multiple comparisons.
