# Supplementary material for: Nuclear myosin VI maintains replication fork stability
Source: Nat Commun. 2023 Jun 24;14:3787. doi: 10.1038/s41467-023-39517-y (PMC10290672; doi:10.1038/s41467-023-39517-y)
Supplement: Supplementary file 7 — Reporting Summary [file 41467_2023_39517_MOESM7_ESM.pdf]

nature portfolio

Corresponding author(s): Helle D. Ulrich and Hans-Peter Wollscheid

Last updated by author(s): May 3, 2023

Reporting Summary

Nature Portfolio wishes to improve the reproducibility of the work that we publish. This form provides structure for consistency and transparency in reporting. For further information on Nature Portfolio policies, see our Editorial Policies and the Editorial Policy Checklist.

Statistics

For all statistical analyses, confirm that the following items are present in the figure legend, table legend, main text, or Methods section.

n/a	Confirmed	
☐	☒	The exact sample size (*n*) for each experimental group/condition, given as a discrete number and unit of measurement
☐	☒	A statement on whether measurements were taken from distinct samples or whether the same sample was measured repeatedly
☐	☒	The statistical test(s) used AND whether they are one- or two-sided<br>*Only common tests should be described solely by name; describe more complex techniques in the Methods section.*
☒	☐	A description of all covariates tested
☐	☒	A description of any assumptions or corrections, such as tests of normality and adjustment for multiple comparisons
☐	☒	A full description of the statistical parameters including central tendency (e.g. means) or other basic estimates (e.g. regression coefficient) AND variation (e.g. standard deviation) or associated estimates of uncertainty (e.g. confidence intervals)
☐	☒	For null hypothesis testing, the test statistic (e.g. *F*, *t*, *r*) with confidence intervals, effect sizes, degrees of freedom and *P* value noted<br>*Give P values as exact values whenever suitable.*
☒	☐	For Bayesian analysis, information on the choice of priors and Markov chain Monte Carlo settings
☒	☐	For hierarchical and complex designs, identification of the appropriate level for tests and full reporting of outcomes
☒	☐	Estimates of effect sizes (e.g. Cohen's *d*, Pearson's *r*), indicating how they were calculated

Our web collection on statistics for biologists contains articles on many of the points above.

Software and code

Policy information about availability of computer code

Data collection	We used Leica Application Suite X version 3.7.5.24914 for widefield microscopy and Fusion 1.1.0.1 software for confocal microscopy.<br>For western blot based data collection, we used Image Studio version 3.1 or Fusion Capt Advance Fx7 17.03 software<br>For SPR data collection, we used Biacore X100 Control Software version 2.0.2
Data analysis	For data analysis, we used Biacore X100 Evaluation version 2.0.2, Image J 1.53t, GraphPad Prism 8.4.0 (538) and MaxQuant version 1.5.2.8<br>MS data visualization and statisical analyses were performed using the R software environment version 4.2.1<br>Statistical significance was calculated using a moderated t-test (limma package)<br>GO term analysis (biological process) was performed using EnrichR (version 2021)

For manuscripts utilizing custom algorithms or software that are central to the research but not yet described in published literature, software must be made available to editors and reviewers. We strongly encourage code deposition in a community repository (e.g. GitHub). See the Nature Portfolio guidelines for submitting code & software for further information.

Data

Policy information about availability of data

All manuscripts must include a data availability statement. This statement should provide the following information, where applicable:

- Accession codes, unique identifiers, or web links for publicly available datasets
- A description of any restrictions on data availability
- For clinical datasets or third party data, please ensure that the statement adheres to our policy

Parent ion and MS2 spectra were searched against a reference proteome database containing human protein sequences obtained from UniProtKB (HUMAN_2016_05) using the Andromeda search engine.
The mass spectrometry-based proteomics data have been deposited to the ProteomeXchange consortium via the PRIDE partner repository with the data set identifier PXD035394.
Source data are provided with this paper.
Custom codes used for the preparation of volcano and GO term plots as well as the Image J-based quantifications are available on GitHub via the following link: https://github.com/helle-ulrich-lab/myosinVI-replication-fork-stability

Human research participants

Policy information about studies involving human research participants and Sex and Gender in Research.

Reporting on sex and gender	n/a
Population characteristics	n/a
Recruitment	n/a
Ethics oversight	n/a

Note that full information on the approval of the study protocol must also be provided in the manuscript.

Field-specific reporting

Please select the one below that is the best fit for your research. If you are not sure, read the appropriate sections before making your selection.

☒ Life sciences ☐ Behavioural & social sciences ☐ Ecological, evolutionary & environmental sciences

For a reference copy of the document with all sections, see nature.com/documents/nr-reporting-summary-flat.pdf

Life sciences study design

All studies must disclose on these points even when the disclosure is negative.

Sample size	Sample size for all experiments is shown in the respective Figures and are in the range of 80-400 fibers or nuclei analysed per sample. Fiber assays and PLA assays were performed in at least three independent replicates, as indicated. Sample size was chosen to obtain statistical power and lies in the same range compared to previous publications containing fiber assays:<br>- Vujanovic et al., Mol Cell, DOI: 10.1016/j.molcel.2017.08.010<br>- Lemacon et al., Nat Commun., DOI: 10.1038/s41467-017-01180-5<br>- Porebski et al., iScience, DOI: 10.1016/j.isci.2019.10.010
Data exclusions	No data were excluded from the analyses.
Replication	All data were replicated at least two times, Fiber and Proximity ligation assays at least three times. All experiments were reproduced.
Randomization	Experiments were not randomized because we look at individual DNA replication molecules or analyze individual cells from an asynchronous cell populations. Therefore, randomization or covariates management were not necessary for the analyses we performed. All images for PLA, IFs and Fiber assays were randomly taken.
Blinding	The authors were blinded for DNA fiber assay analyses. All other analyses were not blinded due to the intrinsic unbiased nature of respective assays/analyses. For PLA and sIRF assays, we used an Image J - based code for analyses.

Reporting for specific materials, systems and methods

We require information from authors about some types of materials, experimental systems and methods used in many studies. Here, indicate whether each material, system or method listed is relevant to your study. If you are not sure if a list item applies to your research, read the appropriate section before selecting a response.

Materials & experimental systems

n/a	Involved in the study
☐	☒ Antibodies
☐	☒ Eukaryotic cell lines
☒	☐ Palaeontology and archaeology
☒	☐ Animals and other organisms
☒	☐ Clinical data
☒	☐ Dual use research of concern

Methods

n/a	Involved in the study
☒	☐ ChIP-seq
☒	☐ Flow cytometry
☒	☐ MRI-based neuroimaging

Antibodies

Antibodies used

Primary antibodies:
GFP (clone 7.1/13.1) mouse monoclonal (11814460001, Roche), Myosin VI (clone MUD19) mouse monoclonal (M0691, Merck), Myosin VI rabbit polyclonal (via Simona Polo, IFOM, Milan (Italy), Wollscheid et al., NSMB 2016), WRNIP1 rabbit polyclonal (A301-389A-T, Bethyl Laboratories), WHIP (clone A-8) mouse monoclonal (sc-376438, Santa Cruz Biotechnology), DNA-PKcs rabbit polyclonal (4602, Cell Signaling Technology), 53BP1 rabbit polyclonal (NB100-304, Novus Biologicals), SMC1 rabbit polyclonal (A300-055A, Bethyl Laboratories), VCP (clone 7F3) rabbit monoclonal (2649, Cell Signaling Technology), MCM7 (clone D10A11) mouse monoclonal (3735, Cell Signaling Technology), Rad18 (clone 79B1048) mouse monoclonal (12007, Abcam), Rad21 (clone D5Y8S) rabbit monoclonal (12673, Cell Signaling Technology), RFC4 mouse polyclonal (2627, Abcam), PCNA (clone PC10) mouse monoclonal (via Bořivoj Vojtěšek, Masaryk Memorial Cancer Institute in Brno (Czech Republic), Waseem and Lane, J Cell Sci. 1990), PCNA rabbit polyclonal (18197, Abcam), BRCA2 (clone 2B) mouse monoclonal (OP95, Merck), Rad51 (clone D4B10) rabbit monoclonal (8875, Cell Signaling Technology), HLTF (clone ART2) rabbit polyclonal (via Alexandra Belayew, UMONS in Mons (Belgium), Debauve, Mol Cancer 2006), SMARCAL1 (clone A-2) mouse monoclonal (376377, Santa Cruz Biotechnology), ZRANB3 rabbit polyclonal (A303-033A, Bethyl Laboratories), Biotin (clone Hyb-8) mouse monoclonal (ab201341, Abcam), BrdU (clone B44) (IdU) mouse monoclonal (347580, BD Biosciences), BrdU (clone BU1/75 (ICR1)) (CldU) rat monoclonal (ab6326, Abcam), phospho-Chk1 (Ser345) rabbit polyclonal (2341, Cell Signaling Technology), Chk1 (clone 2G1D5) mouse monoclonal (2360, Cell Signaling Technology), phospho-Chk 1 (Ser317) rabbit polyclonal (2344, Cell Signaling Technology), polyHistidine (clone HIS-1) mouse monoclonal (H1029, Merck), beta-actin (clone AC-74) mouse monoclonal (A2228, Merck), ubiquitin (clone P4D1) mouse monoclonal (3936,Cell Signaling Technology)
Secondary antibodies:
IRDye® 680LT donkey anti-rabbit IgG (926-68023, LICOR), IRDye® 680LT donkey anti-mouse IgG (926-68072, LICOR), IRDye® 800CW goat anti-rabbit IgG (926-32211, LICOR), IRDye® 800CW donkey anti-mouse IgG (926-32212, LICOR), Polyclonal goat anti-goat HRP (P044901-2, Dako), Polyclonal goat anti-mouse HRP (P044701-2, Dako), Polyclonal goat anti-rabbit HRP (P044801-2, Dako), Goat anti-Rat IgG (H+L) Secondary Antibody, Alexa Fluor 488 (A-11006, Thermo Fisher Scientific), Goat anti-Mouse IgG (H+L) Secondary Antibody, Alexa Fluor 647 (A-21236, Thermo Fisher Scientific), Goat anti-Rabbit IgG (H+L) Cross-Adsorbed, Alexa Fluor 647 (A-21244, Thermo Fisher Scientific)

Validation

The antibodies against BrdU (CldU) and BrdU (IdU) are routinely used for DNA fiber spreading assay.
Specificities of the antibodies against Myosin VI, WRNIP1, BRCA2, SMARCAL1, ZRANB3, HLTF and Rad51 were confirmed by protein knockdown with specific siRNA for the corresponding target.
Specificity of the antibodies against GFP was confirmed by overexpression of GFP-tagged proteins.
Specificity of the antibodies against polyHistidine was confirmed by blotting against a purified recombinant His-tagged protein.
Specificities of the following antibodies were validated by the manufacturer for western blots as indicated on manufacturer website (see below): Myosin VI (clone MUD19), GFP (clone 7.1/13.1), WRNIP1 rabbit polyclonal, WHIP (clone A-8), DNA-PKcs rabbit polyclonal, 53BP1 rabbit polyclonal, SMC1 rabbit polyclonal, VCP (clone 7F3), MCM7 (clone D10A11), Rad18 (clone 79B1048), Rad21 (clone D5Y8S), RFC4 mouse polyclonal, BRCA2 (clone 2B), SMARCAL1 (clone A-2), ZRANB3 rabbit polyclonal, Biotin (clone Hyb-8), phospho-Chk1 (Ser345) rabbit polyclonal, Chk1 (clone 2G1D5), phospho-Chk 1 (Ser317) rabbit polyclonal, polyHistidine (clone HIS-1), ubiquitin (clone P4D1) and beta-actin (clone AC-74).
Specificities of the following antibodies were validated by the manufacturer for immunofluorescence analyses as indicated on manufacturer website (see below): PCNA rabbit polyclonal, WHIP (clone A-8) and Biotin (clone Hyb-8).
BrdU (CldU) rat (ab6326, Abcam): https://www.abcam.com/brdu-antibody-bu175-icr1-proliferation-marker-ab6326.html
BrdU (IdU) mouse (347580, BD Biosciences): https://www.bdbiosciences.com/en-eu/products/reagents/flow-cytometry-reagents/clinical-discovery-research/single-color-antibodies-ruo-gmp/purified-mouse-anti-brdu.347580
GFP (clone 7.1/13.1) mouse monoclonal (11814460001, Roche): https://www.sigmaaldrich.com/DE/de/product/roche/11814460001
Myosin VI (clone MUD19) mouse monoclonal (M0691, Merck): https://www.sigmaaldrich.com/DE/de/product/sigma/m0691?gclid=EAIaIQobChMIo-y64vvY_gIVh-53Ch0JTw0pEAAYASAAEgIjvfD_BwE&gclsrc=aw.ds
WRNIP1 rabbit polyclonal (A301-389A-T, Bethyl Laboratories): https://www.biomol.com/de/produkte/antikoerper/primaerantikoerper/allgemein/anti-wrnip1-a301-389a-t
WHIP (clone A-8) mouse monoclonal (sc-376438, Santa Cruz Biotechnology): https://www.scbt.com/p/whip-antibody-a-8
DNA-PKcs rabbit polyclonal (4602, Cell Signaling Technology): https://www.cellsignal.com/products/primary-antibodies/dna-pkcs-antibody/4602
53BP1 rabbit polyclonal (NB100-304, Novus Biologicals): https://www.novusbio.com/products/53bp1-antibody_nb100-304
SMC1 rabbit polyclonal (A300-055A, Bethyl Laboratories): https://www.biomol.com/de/produkte/antikoerper/primaerantikoerper/allgemein/anti-smc1-a300-055a-t?number=A300-055A
VCP (clone 7F3) rabbit monoclonal (2649, Cell Signaling Technology): https://www.cellsignal.com/products/primary-antibodies/vcp-7f3-rabbit-mab/2649
MCM7 (clone D10A11) mouse monoclonal (3735, Cell Signaling Technology): https://www.cellsignal.com/products/primary-

antibodies/mcm7-d10a11-xp-rabbit-mab/3735
Rad18 (clone 79B1048) mouse monoclonal (12007, Abcam): https://www.abcam.com/products/primary-antibodies/rad18-antibody-79b1048-ab12007.html
Rad21 (clone D5Y8S) rabbit monoclonal (12673, Cell Signaling Technology): https://www.cellsignal.com/products/primary-antibodies/rad21-d5y8s-rabbit-mab/12673
RFC4 mouse polyclonal (2627, Abcam): https://www.abcam.com/products/primary-antibodies/rfc4-antibody-ab2627.html
PCNA rabbit polyclonal (18197, Abcam): https://www.abcam.com/products/primary-antibodies/pcna-antibody-ab18197.html
BRCA2 (clone 2B) mouse monoclonal (OP95, Merck): https://www.merckmillipore.com/DE/de/product/Anti-BRCA2-Ab-1-Mouse-mAb-2B,EMD_BIO-OP95
SMARCAL1 (clone A-2) mouse monoclonal (376377, Santa Cruz Biotechnology): https://www.scbt.com/p/smarcal1-antibody-a-2
ZRANB3 rabbit polyclonal (A303-033A, Bethyl Laboratories): https://www.biomol.com/de/produkte/antikoerper/primaerantikoerper/allgemein/anti-zranb3-e-ab-65632.60?fs=276103915
Biotin (clone Hyb-8) mouse monoclonal (ab201341, Abcam): https://www.abcam.com/products/primary-antibodies/biotin-antibody-hyb-8-ab201341.html
phospho-Chk1 (Ser345) rabbit polyclonal (2341, Cell Signaling Technology): https://www.cellsignal.com/products/primary-antibodies/phospho-chk1-ser345-antibody/2341
Chk1 (clone 2G1D5) mouse monoclonal (2360, Cell Signaling Technology): https://www.cellsignal.com/products/primary-antibodies/chk1-2g1d5-mouse-mab/2360
phospho-Chk 1 (Ser317) rabbit polyclonal (2344, Cell Signaling Technology): https://www.cellsignal.com/products/primary-antibodies/phospho-chk1-ser317-antibody/2344
polyHistidine (clone HIS-1) mouse monoclonal (H1029, Merck): https://www.sigmaaldrich.com/DE/de/product/sigma/h1029
beta-actin (clone AC-74) mouse monoclonal (A2228, Merck): https://www.sigmaaldrich.com/DE/de/product/sigma/a2228
ubiquitin (clone P4D1) mouse monoclonal (3936,Cell SignalingTechnology): https://www.cellsignal.com/products/primary-antibodies/ubiquitin-p4d1-mouse-mab/3936

Eukaryotic cell lines

Policy information about cell lines and Sex and Gender in Research

Cell line source(s)	U2OS FlpIn cells were kindly provided by Lorenza Penengo (Raso 2020, JBC, DOI: 10.1083/jcb.202002175), HeLa cells used for the identification of myosin VI interactors by mass spectrometry were obtained from Cell Services, Cancer Research UK London Research Institute. The original commercial source could not be verified. HEK293T were purchased from Sigma Aldrich (Merck), Cat#12022001-1VL.
Authentication	All cell lines show typical morphological characteristics when compared to images and information availabe on ATCC. No further authentication was done.
Mycoplasma contamination	All cell lines were tested negative for Mycoplasma.
Commonly misidentified lines (See ICLAC register)	no commonly misidentified cell lines were used in this study.
